# Preparation, In Vitro and In Vivo Evaluation of Nanoemulsion In Situ Gel for Transnasal Delivery of Traditional Chinese Medicine Volatile Oil from *Ligusticum sinense* Oliv.cv. Chaxiong

**DOI:** 10.3390/molecules27217644

**Published:** 2022-11-07

**Authors:** Chunhui Huang, Canjian Wang, Wenliu Zhang, Tao Yang, Mingyan Xia, Xiaomeng Lei, Ying Peng, Yuhuan Wu, Jianfang Feng, Dongxun Li, Guosong Zhang

**Affiliations:** 1 Key Laboratory of Modern Preparation of TCM, Ministry of Education, Jiangxi University of Chinese Medicine, Nanchang 330006, China; 2 Wuzhou Traditional Chinese Medicine Hospital, Wuzhou 543001, China; 3 College of Pharmacy, Guangxi University of Chinese Medicine, Nanning 530200, China; 4 National Engineering Research Center of Chinese Medicine Solid Preparation Manufacturing Technology, Nanchang 330006, China; 5 College of Chinese Medicine and Life Science, Jiangxi University of Chinese Medicine, Nanchang 330004, China

**Keywords:** volatile of *Ligusticum sinense* Oliv.cv. Chaxiong, nanoemulsion, in situ gel, nasal administration

## Abstract

Ischemic stroke is a difficult-to-treat brain disease that may be attributed to a limited therapeutic time window and lack of effective clinical drugs. Nasal–brain administration is characterized by low systemic toxicity and is a direct and non-invasive brain targeting route. Preliminary studies have shown that the volatile oil of Chaxiong (VOC) has an obvious anti-ischemic stroke effect. In this work, we designed a nanoemulsion thermosensitive in situ gel (VOC-NE-ISG) loaded with volatile oil of Chaxiong for ischemia via intranasal delivery to rat brain treatment of cerebral ischemic stroke. The developed VOC-NE-ISG formulation has a suitable particle size of 21.02 ± 0.25 nm and a zeta potential of −20.4 ± 1.47 mV, with good gelling ability and prolonged release of the five components of VOC. The results of in vivo pharmacokinetic studies and brain targeting studies showed that intranasal administration of VOC-NE-ISG could significantly improve the bioavailability and had excellent brain-targeting efficacy of nasal-to-brain delivery. In addition, the results of pharmacodynamics experiments showed that both VOC-NE and VOC-NE-ISG could reduce the neurological deficit score of model rats, reducing the size of cerebral infarction, with a significant effect on improving ischemic stroke. Overall, VOC-NE-ISG may be a promising intranasal nanomedicine for the effective treatment of ischemic stroke.

## 1. Introduction

Cerebral stroke, commonly known as “stroke”, is an acute cerebrovascular disease caused by sudden rupture or blockage of blood vessels in the brain, resulting in normal blood flow into the brain and brain tissue damage. It has the characteristics of high rates of disability, fatality, and recurrence, and is one of the most lethal diseases in the world [1]. Stroke includes ischemic and hemorrhagic stroke. About 80% of these strokes are ischemic in nature, also known as ischemic stroke or cerebral infarction, and are caused by a transient or permanent reduction in cerebral blood flow (CBF), which is limited to the main areas of cerebral arteries, such as middle cerebral artery occlusion (MCAO). Blockage of blood vessels results in a lack of oxygen and energy in brain tissue, leading to inflammation that can lead to irreversible tissue damage [2]. The clinical manifestations are mainly characterized by sudden dizziness, unconsciousness, or the sudden appearance of slanted eyes, hemiplegia, and intellectual disability. The study reported that it may be salvageable if appropriate treatment is administered within a specified golden therapeutic window. At present, reperfusion and neuroprotection were mainly developed to treat ischemic stroke. The latter treatment strategy was to re-drain blood vessels with thrombolytic drugs or mechanical devices to restore perfusion as quickly as possible. However, when cerebral ischemia persists for a long time, treatment to restore blood flow and reperfusion can lead to brain damage, cerebral edema, cerebral hemorrhage, and neuronal death, etc., known as cerebral ischemia-reperfusion injury (CIRI) [3]. At present, the drugs used in the treatment of ischemic stroke include thrombolytic drugs (t-PA, urokinase, etc.), antithrombotic drugs, antiplatelet drugs, fibrinolytic drugs, microcirculation-improving drugs, etc. t-PA (tissue plasminogen activator) was the first drug approved by FDA for the treatment of stroke in 1996, but due to the strict treatment window of ischemic stroke (within 3 h), many patients could not be treated, and there was a high risk of intracranial hemorrhage rate. There are many drugs in the domestic market, such as nimodipine, flunarizine, yatan, karan and ginkgo biloba extract, which are used to improve cerebral circulation, brain metabolism and brain function. Although they all have certain therapeutic effects, their efficacy in the treatment of stroke cannot be fully affirmed.

At present, the representative clinical drug is butylphthalide (NBP) for the treatment of ischemic stroke, a class 1 new drug developed in China. Butylphthalide is widely found in Umbelliferae, such as Chuanxiong, Chaxiong, and so on. Therefore, other more widely available treatment options are urgently needed. Chaxiong, also known as Fuxiong, is the dried rhizome of *Ligusticum sinense* Oliv.cv. Chaxiong [4]. Chaxiong is mainly produced in the Jiujiang area of Jiangxi Province, where it has a long history of cultivation and medicinal use. Chaxiong is warm in nature and acrid in taste and has the effects of expelling wind and relieving pain, promoting qi and promoting blood circulation. Chaxiong volatile oil (volatile oil from *Ligusticum sinense* Oliv.cv. Chaxiong, VOC) is the volatile oil obtained from the rhizome of Chaxiong by steam POPE molecular distillation. Luo Yongming et al. [5] used the GC-MS technique to separate and identify the volatile oil of Chaxiong and identified 21 chemical components, including five phthalide components, Yangchuanxiong lactone A, n-butylphthalide, new Cnidolide, Z-ligustilide and butenylphthalide, which accounted for about 90% of the total volatile oil. The study showed that the volatile oil of Chaxiong has the effects of antagonizing the neurotoxicity of glutamic acid, inhibiting the heartbeat, enhancing coronary blood flow and dilating blood vessels, etc. It can be used for cerebral thrombosis and stroke and cerebral edema caused by focal cerebral ischemia [6].

The research group conducted preliminary pharmacodynamic research on the volatile oil of Chaxiong in the early stage, and it has been preliminarily confirmed that the volatile oil of Chaxiong is the main pharmacodynamic part of the anti-ischemic stroke of Chaxiong. The study found that the volatile oil of Chaxiong has a good protective effect on the primary cultured rat neurons mediated by H_2_O_2_ and glutamate and also has a certain protective effect on the rat MCAO reperfusion model. However, the volatile oil of Chaxiong is highly volatile, insoluble in water and has low oral bioavailability, which limits its clinical application in ischemic stroke.

The inability of therapeutic agents to cross the blood–brain barrier (BBB) and blood–cerebrospinal fluid barrier (CSF) represents a major obstacle in the treatment of ischemic stroke. One possibility to overcome this hurdle is to use novel drug-delivery systems to deliver drugs to the brain. Intranasal delivery routes can enhance drug delivery to the brain through the use of appropriate vehicle systems (e.g., liposomes, nanoparticles, nanoemulsions, etc.), thereby enhancing CNS permeability to the drug and allowing sustained drug delivery. Mukesh Kumar et al. prepared risperidone-loaded nanoemulsions (RNE) and muco-adherent nanoemulsions (RMNE) for the treatment of schizophrenia, and their radiolabel-labeled and gamma scintigraphy imaging studies demonstrated that muco-adherent risperidone nanoemulsions provided rapid and efficient intranasal drug delivery to the brain. As a non-invasive topical-drug-delivery method, the nasal–brain pathway not only bypasses the blood–brain barrier but also avoids the first-pass effect of the liver. Nasal administration has gradually become one of the research hotspots of drug administration routes due to its advantages of no hepatic first-pass metabolism, fast drug absorption, and high bioavailability. As a local or systemic absorption drug, the nasal mucosa has the advantages of relatively large drug absorption surface area, abundant capillaries and lymphatic capillaries, and abundant total blood flow. These advantages enable the nasal route to significantly increase drug absorption, significantly increase the amount of drug delivered to the brain, and improve the therapeutic effect of brain diseases. Additionally, many studies have shown that drugs can be effectively transferred directly from the nasal cavity to the brain via the nasal route. Therefore, it is considered to develop the volatile oil of Chaxiong into a preparation for intranasal administration, which is used for intranasal administration to transport the drug to the brain through the nose.

In recent years, drug delivery by nanoemulsion has proven to be a promising strategy for improving the bioavailability of lipophilic drugs and has received increasing attention from researchers [7,8]. Nanoemulsion is a thermodynamically, kinetically stable, isotropic transparent dispersion system composed of water phase, oil phase, emulsifier and co-emulsifier [9]. Nanoemulsion has the advantages of small particle size, high solvent capacity and excellent stability. Nanoemulsions can not only increase the solubility of poorly soluble drugs but also carry drugs with different solubilities, achieving synergistic effects between drugs and prolonging the effect. They improve the bioavailability of the drug and play a sustained-release and targeted role [10]. The emulsifier and co-emulsifier in the nanoemulsion can reduce the interfacial tension between the oil and water of the emulsion, and at the same time, reducing the particle size to the nanometer level can reduce the aggregation and flocculation of droplets, thereby significantly improving the stability of the emulsion. However, the rapid clearance of numerous cilia on the nasal mucosa limits intranasal drug delivery. The direct nasal administration of nanoemulsion may cause leakage or irritation to the nasal mucosa. Therefore, the use of nasal nanoemulsion in situ gel in this paper can effectively avoid or reduce the clearance of nasal cilia. The volatile oil of Chaxiong is embedded in the nanoemulsion, which can increase the solubility of the drug, improve the stability of the volatile oil, and prevent the drug from being degraded before reaching the action site. The in situ gel can prolong the retention time of the drug in the nasal mucosa, promote the dissolution and penetration of drugs in the nasal mucosa, avoid drug leakage, reduce drug drips into the back of the throat, and improve bioavailability [11].

Studies have shown that Chaxiong volatile oil was a potential anti-ischemic stroke drug, and its further application is limited due to its instability and low water solubility, which seriously affect its accumulation in the brain. The aim of this study is to develop a brain drug-delivery system loaded with Chaxiong volatile oil nanoemulsion in situ gel for the effective treatment of ischemic stroke by direct nasal–brain administration. In this study, a temperature-sensitive nanoemulsion in situ gel formulation (VOC-NE-ISG) loaded with volatile oil of Chaxiong was developed by phase transformation and “cold” methods. In addition, this study also aimed to evaluate VOC-NE-ISG physicochemical properties, pharmacokinetic studies, and in vivo anti-ischemic stroke effects, as well as preliminary brain targets for ischemic stroke therapy by nasal-to-brain administration towards ability.

## 2. Results and Discussion

### 2.1. Screening of Nanoemulsion Formulations

#### 2.1.1. Screening of Cosurfactant

Because cosurfactants are amphiphilic, they can accumulate in the interface layer and reduce the interfacial tension, which enables them to increase the fluidity of the interface film and penetrate the single layer of the surfactant [12]. It can be seen from Figure S3 (Supplementary Materials) that the solubility of the five components of the volatile oil of chaxiong in ethanol absolute was the highest, and the solubility in PEG400, propylene glycol, and n-butanol was relatively high, while it was almost immiscible in glycerol. Therefore, in this experiment, anhydrous ethanol was selected as the co-surfactant to further prepare the volatile oil of Chaxiong nanoemulsion.

#### 2.1.2. Screening of Surfactant

In general, non-ionic surfactants have high surface activity, good solubility, and are less toxic and irritating than ionic surfactants, so all surfactants and cosurfactants used in this study were selected as non-ionic type. Emulsifiers promote the formation of nanoemulsions by reducing interfacial tension, thereby promoting the free energy loss associated with nanodroplet formation [13]. An emulsifier is a kind of surface-active agent, which can improve the surface tension between the various constituent phases in the emulsion body and form a uniform emulsion body. Each non-aqueous component has a desired HLB value; the emulsification characteristics of emulsifier depend on its hydrophilic and oleophilic equilibrium value (HLB value). The higher the HLB value, the stronger the hydrophilicity, and vice versa. The optimum HLB value for the emulsifier is close to the HLB value required for the oil. When several emulsifiers are used in combination, the HLB value of the composite emulsifier has the additivity according to the mass fraction.

According to the results in Table S4 (Supplementary Materials), it can be concluded that when RH40 is used as an emulsifier, a nanoemulsion can be formed, and the appearance is clear and transparent, the particle size is 23.11 ± 0.12 nm, and the PDI is 0.474 ± 0.23. The HLB value of O/W type nanoemulsion is generally 8~18, while the HLB value of Span 80 is 4.3, which is not suitable for forming O/W type nanoemulsion. VOC has high solubility in surfactant cremoophor RH 40, and its HLB value is between 14 and 16. Therefore, RH40 was selected as an emulsifier for the study of nanoemulsions.

#### 2.1.3. Pseudo-Ternary Phase Diagram

The pseudo ternary phase diagram was developed to determine the concentration range of components forming nano emulsion. It can be seen from Figure S4 (Supplementary Materials) that when the ratio of oil to Smix is 9:1, 8:2, and 7:3, 6:4, 5:5, 4:6, the prepared emulsion system is a turbid yellow-white emulsion. The test results show that the maximum area of O/W nanoemulsion region was observed when Km was 2:1.

#### 2.1.4. Optimization of VOC-Nanoemulsion

According to the experimental results, Design Expert 8.0.6 software (Stat-Ease Inc., Minneapolis, MN, USA) as used to perform multiple linear regression fitting on the data to analyze the main effect, interaction effect and quadratic effect of three independent variables on three responses. Visual analysis and ANOVA were performed on the test results to screen out the optimal nanoemulsion prescription. The results are shown in Table 1. The ANOVA results of the three response values obtained by regression analysis are shown in Table S5 (Supplementary Materials). The *p* value of lack of fit of the three response values was >0.05, indicating that the lack of fit test was insignificant, implying that there was a significant model correlation between the independent variables and the response values. The polynomial equations for size, PDI and ZP were as follows:

$$Y1=30.52+20.57A+1.12B-11.3C-13.9\mathrm{AB}+10.06\mathrm{AC}+9.64\mathrm{BC}+2.94A^{2}+15.87B^{2}+20.69C^{2}\;Y2=0.31+0.099A-0.061B-0.047C-0.032\mathrm{AB}+0.005\mathrm{AC}+0.025\mathrm{BC}-0.09A^{2}+0.02B^{2}+0.16C^{2}\;Y3=-13.3-0.31A+0.92B-0.26C-1.53\mathrm{AB}-1.08\mathrm{AC}-1\mathrm{BC}+0.55A^{2}-0.57B^{2}-0.37C^{2}$$

The analysis of variance (ANOVA) was performed on the above particle size model. The analysis shows that the F value of the model is 8, the *p* value is 0.006, reaching a very significant level (*p* < 0.01), and the lack of fit (Lack of Fit) *p* value is 0.0539 > 0.05, indicating that the model is less interfered by other factors, and the model fits well. The results of the analysis of variance of the potential model are shown in Table S5 (Supplementary Materials). The *p* value of the model is 0.0129, indicating that the model has reached a significant level (*p* < 0.05), and the *p* value of the lack-of-fit term is >0.05, indicating that the model fits well.

According to the above data and the model fitting results, using Design-Expert 8.0.6 software (Stat-Ease Inc., Minneapolis, MN, USA) for mapping, we can obtain the 3D ratio of oil ratio (A), Smix ratio (B), stirring time (C) to response value, particle size and potential. The effect surface map and contour map are shown in Figure S5 (Supplementary Materials) and Figure S6 (Supplementary Materials). It can be seen from the results that A (oil phase ratio) and C (stirring time) have a very significant effect on particle size Y1, while B (Smix ratio) has a small effect on particle size Y1, and A and B have a significant interaction effect on the particle size; B (Smix ratio) has a more significant effect on the potential Y2, and A, B, and C all have significant interaction effects. 

RH40 has a good hydrophile lipophilic balance (14~16), which is conducive to the formation of O/W nanoemulsion. In addition, RH40 is generally recognized as safe (GRAS) and can be used as pharmaceutical and food surfactants. After the preparation of nano emulsion, the growth of droplet may occur due to Ostwald maturation or coalescence process, depending on the composition of surfactant and oil. The 3D surface results showed that the increase in the concentration of Smix used in the preparation of NE reduced the average particle size. With the moderate increase in surfactant concentration, the particle size will also decrease significantly, because additional surfactant can stabilize more of the interface area. Because the newly formed interface cannot be completely stabilized under the condition of insufficient surfactant, the coalescence rate of liquid drops is very significant.

Using the process optimization function of Design-Expert 8.0.6 software, the smallest particle size and the lowest potential are substituted into the analog linear equation so that the particle size and potential are as small as possible and the best prescription process can be predicted: the ratio of oil (A) was 6.67%, the ratio of Smix (B) was 26.67%, and stirring time (C) was 1.27 h. The predicted particle size was 21.38 nm and the zeta potential was −21.33 mV. The test results are shown in Table S6 (Supplementary Materials). It was verified that the particle size of three batches of VOC-NE was 21.02 ± 0.17 nm, PDI was 0.15 ± 0.02, and zeta potential was −22.87 ± 1.02 mV. The deviation rate of the particle size and zeta potential was 1.71% and 1.72%, respectively. This indicates that the model has a high degree of fit.

### 2.2. Characterization of VOC-NE

#### 2.2.1. Traits and Types

Through observation, it was found that the volatile oil of Chaxiong nanoemulsion was a light yellow, clear liquid with Tyndall phenomenon, with no turbidity, flocculation, stratification, or other phenomena (Figure S7, Supplementary Materials).

According to the diffusion speed of water-soluble dye methylene blue and oil-soluble dye Sudan red III in nanoemulsion, the type of Chaxiong volatile oil nanoemulsion was determined. The identification results of the staining method are shown in Figure S8 (Supplementary Materials). The water-soluble dye methylene blue diffuses very quickly in the Chaxiong volatile oil nanoemulsion. After a period of time, the nanoemulsion is dyed blue by methylene blue, while Sudan red III cannot diffuse in the nanoemulsion, which settles directly at the bottom. This indicates that the VOC-NE formulation in this experiment was an oil-in-water nanoemulsion.

The conductivity measurement depends on the poor conductivity of oil compared to water. In the water-in-oil nanoemulsion with oil as the continuous phase, the conductivity is very low, close to the conductivity of oil. On the contrary, oil-in-water nanoemulsion was close to the conductivity of water with a higher conductivity [14]. Therefore, the conductivity value of the nanoemulsion can help to judge the type of the prepared nanoemulsion. The conductivity of Chaxiong volatile oil nanoemulsion was measured to be 31.62 ± 0.17 µS/cm, the conductivity of water was 10.8 µS/cm, and the conductivity of oil was 0.65 µS/cm. The experimental results show that the VOC-NE was an oil-in-water nanoemulsion. This is consistent with the results of the dye identification test.

#### 2.2.2. Particle Size, PDI and Zeta Potential

For nanoparticle drug-delivery systems, the particle size has a significant effect on the absorption and penetration of the drug or particle. The particle size is a key value for evaluating nanoemulsions. The smaller particle size provides a larger interfacial surface area for drug absorption. PDI reflects the uniformity of the nanoemulsion particle size and can also reflect the stability of nanoemulsion. The smaller the polydispersity coefficient, the more uniform the size of the prepared nanoemulsion droplets and the more stable the prepared nanoemulsion [15].The low surface charge may be related to the intrinsic properties of nonionic surfactants. The higher the absolute value of the potential, the higher the surface charge of the droplets, the greater the repulsive force between the droplets, and the more stable the prepared nanoemulsion [11]. The optimized VOC-NE has a particle size of 21.02 ± 0.25 nm, a PDI of 0.150 ± 0.02, and a potential of −22.87 ± 1.02 mV. Figure S9 (Supplementary Materials) shows the particle size distribution and potential distribution of the VOC-NE.

#### 2.2.3. pH, Viscosity, Refractive Index and Transmittance

The pH value of VOC-NE was 4.52 ± 0.02, which is well tolerated in the range of the human nasal pH range (4.5–6.5), so it will not cause nasal irritation when administered intranasally. 

The viscosity of the formulation may affect the absorption of the entire nasal mucosa. The formulation with high viscosity tends to stay longer on the mucosa and increase the stay time in the nasal cavity. However, higher viscosity may also lead to delayed drug release from the carrier to the mucosal surface. These formulations have Newtonian rheological properties because they have a constant viscosity. The viscosity of the volatile oil of Chaxiong nanoemulsion was 32 ± 0.5 mPa·s, which is low and very close to the viscosity of water. The low viscosity value ensures easy handling, packaging, and trouble-free administration of the formulation.

The refractive index (RI) is a characteristic of a medium to light volatile oil, which is used to characterize the isotropy of nanoemulsions. The refractive index of the volatile oil of Chaxiong nanoemulsion was measured by Abbe refractometer to evaluate the isotropy of the nanoemulsion. The results show that no significant difference was observed in the RI values between the various VOC-NE formulations, which indicated that the nanoemulsion formulations were chemically stable and remained isotropic in nature.

The percent transmittance is the net value of each component of the nanoemulsion, indicating the isotropic nature of the formulation. The experimental results showed that the %T values of all the VOC-NE were 99.63% ± 0.40, between 97% and 100%, indicating that the volatile oil nanoemulsions of Chaxiong were optically transparent.

#### 2.2.4. Result of TEM

**Figure 1:**
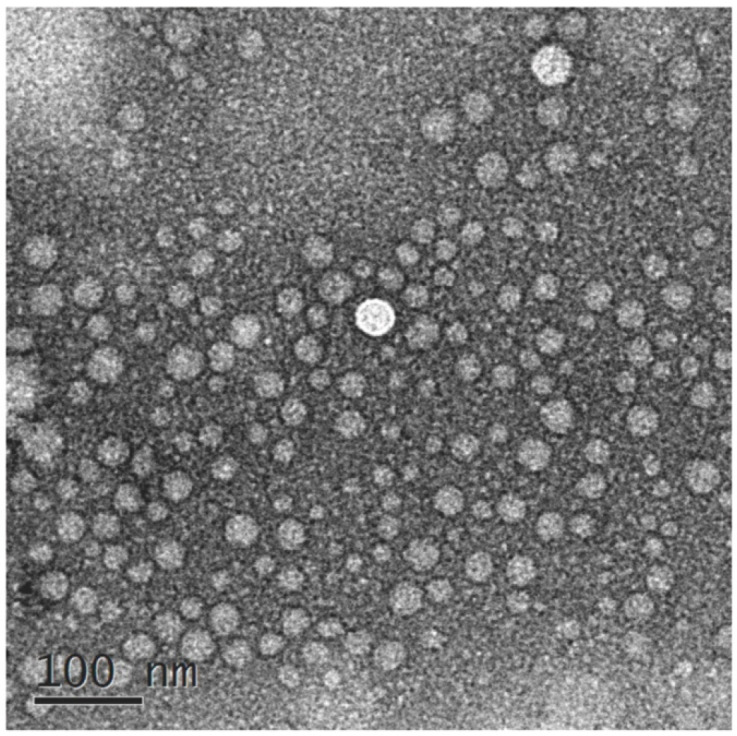
TEM images of VOC-NE.

The morphology of VOC-NE formulation was characterized by TEM. Figure 1 shows TEM images of the optimized VOC-NE formulation. The nanoemulsion droplets were spherical and the particle size ranged from 20 nm to 50 nm. The TEM results of particle size analysis are similar to the average particle size obtained by particle size analyzer.

#### 2.2.5. Result of Stability Studies

The results of the centrifugation stability test showed that the appearance of the VOC-NE did not change after centrifugation, and no stratification, demulsification, or other phenomena occurred, and it was still a light-yellow, clear liquid. The results of freeze–thaw stability showed that after four freeze–thaw cycles, the VOC-NE did not appear display delamination or demulsification, the particle size ranged from 18 to 31 nm, and the PDI was 0.14 to 0.20, indicating that the VOC-NE formulation has a good freeze–thaw stability. The heating–cooling stability results showed that the VOC-NE did not display delamination or demulsification after four heating and cooling cycles, the particle size ranged from 18 to 35 nm, and the PDI was 0.15 to 0.20, which indicated that the VOC-NE has good freeze–thaw stability. In summary, the volatile oil of Chaxiong nanoemulsion has a good centrifugation, freeze–thaw and heating–cooling stability.

### 2.3. Screening of VOC-NE-ISG Gel Framework Materials

#### 2.3.1. The Effect of P407 on the Gelling Temperature of In Situ Gels

The results of the gelation temperature are shown in Figure S10 (Supplementary Materials). It can be seen from the figure that the poloxamer 407 solution can undergo phase transformation to form a gel after being heated. Additionally, in the range of P407 concentration from 16% to 24%, the gelation temperature of in situ gels decreased gradually with the increase in P407 concentration.

#### 2.3.2. The Effect of P188 on the Gelling Temperature of In Situ Gels

It can be seen from Figures S10 and S11 (Supplementary Materials) that the concentrations of P407 and P188 have a greater influence on the gelation temperature of the in situ gel. The results showed that the gelation temperature of the in situ gel decreased with the increase in the concentration of P407, and increased with the increase in the concentration of P188. This is consistent with literature reports.

#### 2.3.3. Screening Prescription Ratio of VOC-NE-ISG

The previous preliminary experiments found that the gel matrix could not be fully swelled by directly adding VOC-NE. Therefore, the total volume of the solvent was fixed, and the ratio of fixed water and Chaxiong volatile oil nanoemulsion was 1:1. The experimental results show that when the ratio of water and VOC-NE was 1:1, the ratio of P407 was 12%, and the ratio of P188 was 10%, the gelation temperature at this time was 33 °C (Figure S12, Supplementary Materials).

### 2.4. In Vitro Evaluation of VOC-NE-ISG

#### 2.4.1. Measurement of pH and Viscosity of the Gel

The experimental results showed that the three batches of VOC-NE-ISG samples were all pale-yellow, transparent liquids, the pH value was around 5.85, and the pH of the nasal cavity was 4.5–6.5. The viscosity of VOC-NE-ISG at room temperature is about 1799 m·Pa s, and the gel viscosity at physiological temperature is about 4500 m·Pa s. The viscosity of the formulation can ensure prolonged contact and effectively improve the nasal absorption level of drugs and nanoparticles. However, higher viscosity may also result in delayed drug release from the carrier to the mucosal surface.

#### 2.4.2. Measurement of Gelation Temperature and Gelation Time

The temperature of the nasal cavity is generally 29–34 °C. If the gelling temperature of the in situ gel is too high, it may not be converted into a gel after administration. If the gelling temperature is too low, it may not be possible to administer in liquid form. The experimental results show that the gelation temperatures of the three batches of VOC-NE-ISG samples prepared are basically the same, about 33 °C. Figure S13 (Supplementary Materials) shows the liquid–solid transition state of VOC-NE-ISG. As shown in the figure, when the ambient temperature is lower than the gelation temperature, the VOC-NE-ISG has good fluidity; when the ambient temperature is equal to the gelation temperature, the VOC-NE-ISG transforms into an immobile gel state. The gelation time of three batches of VOC-NE-ISG samples was about 52 s.

#### 2.4.3. Determination of Gel Strength

The gel strength measurement results showed that the gel strength of the VOC-NE-ISG was 47~49 s. The two most important metrics for in situ gels are viscosity and strength [16]. Studies have shown that the appropriate viscosity and strength of nasal gel can ensure the long-term retention of the gel at the administration site, increase the drug retention time, improve the absorption efficiency of the nasal cavity, and then improve the transport efficiency of the drug from the nose to the brain.

#### 2.4.4. Determination of Bioadhesion

The mucoadhesion strength of VOC-NE-ISG was (5.69 ± 0.51) × 10^2^ dynes/cm^2^. The stronger the mucoadhesion, the better the ability to prevent the gel solution from flowing out of the nasal cavity and into the nasopharynx.

#### 2.4.5. Determination of Expansion Coefficient and Gel Water Retention Capacity

The volume of the human nasal cavity was limited, so the volume expansion coefficient of the gel after gelation was required to be less than 5% [17]. The experimental results show that the expansion coefficient of VOC-NE-ISG was 1.96%, less than 5%, and there is no obvious volume expansion. Therefore, when the Chaxiong volatile oil nanoemulsion in situ gel is instilled into the nasal cavity, it will not affect the patient’s compliance. The water retention capacity of in situ gels can be used to evaluate the storage and transport stability of gels [18]. The water retention capacity of VOC-NE-ISG was 97.82 ± 0.36%, indicating its good storage and transportation stability.

#### 2.4.6. Rheological Studies

It can be seen from Figure S14A (Supplementary Materials) that the strain (γ) < 1%, G′ > G″, and elasticity dominates, that is, VOC-NE-ISG exhibits a gel structure, when γ > 1%, G′ gradually decreases, and when G′ = G″, the gel structure of VOC-NE-ISG is completely destroyed [19], and then with the gradual increase in γ, G′ gradually decreases, and G″ gradually increases. At this time, G′ < G″, viscosity predominates, showing fluid properties. Therefore, all dynamic oscillation experiments need to control the strain γ within 1%.

Frequency sweeps were used to characterize the time-dependent properties of the samples in the non-destructive deformation range and to characterize the stability of the internal structure of the gel. The frequency scanning of VOC-NE-ISG was performed in the oscillation mode of the rheometer to investigate the stability of the internal structure of the in situ gel. In this experiment, the strain is 1% and the frequency sweep range is 0.1 to 100 Hz for the frequency sweep. The test results are shown in Figure S14B (Supplementary Materials). The test results show that in the scanning range of 0.1 to 100 Hz, G′ is always greater than G″, showing obvious elastic-dominated characteristics, and the variation of G′ is small and stable, and there is no obvious frequency dependence, which indicates that VOC-NE-ISG always has a stable 3D network structure.

Using the rotational mode of the rheometer, the VOC-NE-ISG was scanned at a shear rate (0.1–100 s^−1^) at 25 and 33 °C, respectively, to preliminarily determine the fluid type of the sample. Figure S14C,D (Supplementary Materials) were the change curves of the shear rate (γ′), shear stress (τ) and viscosity (η) of VOC-NE-ISG at 25 °C and 33 °C. It can be seen from Figure S14C,D (Supplementary Materials) that in the continuous shear rheological measurement, the shear stress and shear rate of the in situ gel of Chaxiong volatile oil nanoemulsion showed a nonlinear relationship, and τ gradually increased with the overall change in γ′, which is large, and η has the property of decreasing and leveling off with the increase in γ′, which belongs to pseudoplastic fluid and is a typical characteristic of a hydrophilic polymer system. A higher degree of pseudoplasticity facilitates their smearing on biological surfaces [20]. The test results show that VOC-NE-ISG exhibits stable pseudoplastic fluid characteristics at 25 and 33 °C.

#### 2.4.7. In Vitro Release Studies 

The in vitro release model fitting results of each component of VOC-NE-ISG are shown in Table S7 (Supplementary Materials), and the in vitro release curves are shown in Figure S15 (Supplementary Materials). According to the fitting results of the release model, the release mechanism of the five components in VOC-NE-ISG is closer to the Ritger–Peppas release, 0.45 < *n* < 0.89, and the release mechanism is the combined effect of diffusion and matrix erosion.

#### 2.4.8. Histological Evaluation of Nasal Mucosa

**Figure 2:**
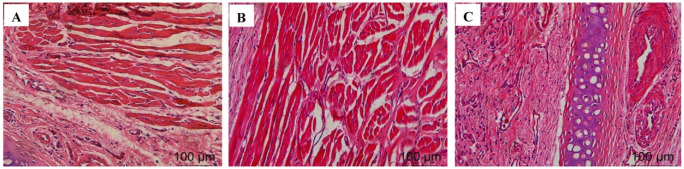
Nasal mucosa pathological section results of VOC-NE-ISG (*n* = 4). (**A**) saline group; (**B**) VOC-NE-ISG group; (**C**) isopropyl alcohol group.

The test results are shown in Figure 2. A large amount of damage and inflammatory cell infiltration appeared in the nasal mucosa of the rats in the isopropanol nasal administration group. The nasal mucosa of the rats in the VOC-NE-ISG group and the normal saline group showed similar microstructures, no cell necrosis was observed, and no significant damage and inflammatory cell infiltration occurred, indicating that the prepared VOC-NE -ISG is safe and has no obvious irritation to the nasal mucosa.

### 2.5. Results of Pharmacokinetic Study

**Figure 3:**
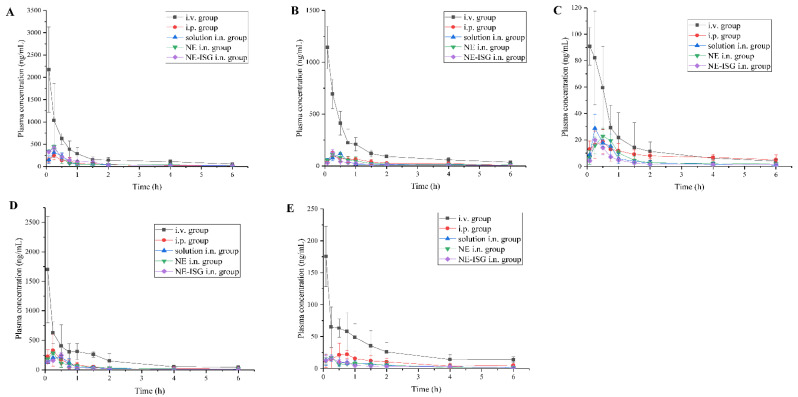
Concentrations of components plasma samples of each group after administration of VOC preparation (*n* = 3, X ± SD). (**A**) Senkyunolide A. (**B**) Neocnidolide. (**C**) n-Butylphthalide. (**D**) Z-ligustilide. (**E**) Butenyl phthalide.

The plasma concentration–time curve was shown in Figure 3, and the main pharmacokinetic parameters were shown in Table 2.

It can be obtained from the experimental results that after oral administration of the volatile oil of Chaxiong to rats, the peak time Tmax of its five components is between 0.21 and 0.50 h, indicating that the oral absorption of the five components is faster. Compared with the intravenous injection group, its peak concentration C_max_ is lower, and the five components of Chaxiong volatile oil are Senkyunolide A, neocnidolide, n-butylphthalide, Z-ligustilide and butenylbenzene. The absolute bioavailability of phthalein was 17.19%, 19.19%, 18.32%, 9.54% and 10.91%, respectively. Compared with the intravenous injection group, the Tmax and t_1/2_ of each component of the volatile oil of Chaxiong were prolonged in the nasal solution group. The absolute bioavailability of ligustilide and butenylphthalide was 26.91%, 25.53%, 26.13%, 21.28% and 23.10%, respectively. Compared with intragastric administration of Chaxiong volatile oil, after preparing Chaxiong volatile oil into a nanoemulsion in situ gel preparation, its peak time Tmax and t1/2 were prolonged, and the degrees of absolute bioavailability of the five components of Chaxiong volatile oil were 42.16%, 40.69%, 34.74%, 29.93% and 31.63%, which were significantly greater than 25%, indicating that the absolute bioavailability of the five components in the VOC-NE-ISG was improved to varying degrees. Compared with the nasal solution group, the T_max_ and t_1/2_ of the VOC-NE group were significantly increased, indicating that the modification of the volatile oil of Chaxiong with nanoemulsion could increase the amount of drug absorption. Compared with the VOC-NE group, the Tmax of the VOC-NE-ISG group was decreased, and the t_1/2_ was significantly increased (*p* < 0.05), indicating that the preparation of nanoemulsion into an in situ gel nasal preparation can significantly increase the retention time of the drug in the nasal cavity, thereby increasing drug absorption and improving bioavailability. Compared with the nasal solution group, the absolute bioavailability of the five components of Chaxiong volatile oil in the VOC-NE-ISG group increased by 1.57, 1.59, 1.33, 1.41 and 1.36 times, respectively, indicating that the volatile oil of Chaxiong was prepared into nanoemulsion in situ coagulation. The glue can significantly improve the bioavailability of each component and increase the transport from the nose to the brain.

### 2.6. Results of Brain Tissue Distribution Studies

**Figure 4:**
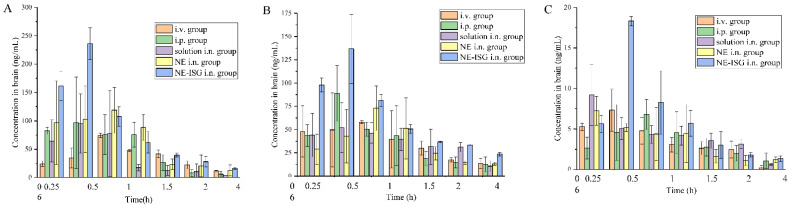
Concentrations of components in brain tissue samples of each group after administration of Chaxiong volatile oil preparation (*n* = 3, X ± SD). (**A**) Senkyunolide A. (**B**) Neocnidolide. (**C**) n-Butylphthalide.

**Figure 5:**
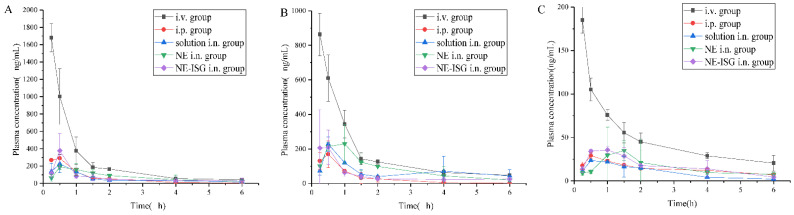
Concentrations of components plasma samples of each group after administration of Chaxiong volatile oil preparation (*n* = 3, X ± SD). (**A**) Senkyunolide A. (**B**) Neocnidolide. (**C**) N-Butylphthalide.

The measured drug concentrations in the rat brain tissue were averaged, and the brain tissue drug concentration–time curves of each group were drawn. The plasma pharmacokinetic parameters and brain tissue pharmacokinetic parameters are shown in Table 3. The brain tissue drug time curve is shown in Figure 4 and the blood drug time curve is shown in Figure 5.

It can be seen from the experimental results that compared with the gavage group and the intravenous injection group, after intranasal administration of VOC-NE-ISG, the Tmax of the drug in the rat brain tissue was significantly reduced, while the t_1/2_, Cmax and AUC_0–t_ were significantly increased, indicating that intranasal administration of VOC-NE-ISG can facilitate drug delivery into the brain. Compared with the nasal solution group, the AUC_0–t_ of the VOC-NE group was significantly increased, indicating that the modification of VOC-NE could increase the transport amount of the drug absorbed through the nose into the brain. The AUC_0–t_ value of the VOC-NE-ISG group was significantly different from that of the nasal solution group (*p* < 0.05), indicating that the in situ gel can prolong the retention time of the drug in the nasal cavity and increase the absorption of the drug.

According to the experimental results, the order of the brain targeting index in each group was: NE-ISG group > NE group > nasal solution group > intravenous injection group. The BTIs of the three components Senkyunolide A, neocnidolide and n-butylphthalide in the intranasal administration of VOC-NE-ISG were 1.73, 1.56, and 2.01, respectively, all greater than 1.0, indicating that VOC-NE -ISG formulations have obvious brain-targeting properties. Additionally, the larger the BTI, the more obvious its brain targeting.

### 2.7. Results of Pharmacodynamic Study

#### 2.7.1. Results of Neurological Deficit Score

In this experiment, VOC-NE and VOC-NE-ISG were administered intranasally for evaluating the therapeutic efficacy in MCAO rats. The cerebral infarct size and neurological score were used to assess the therapeutic efficacy of the prepared formulations. In this experiment, the rat middle artery occlusion model was established by the suture method. This model is consistent with human cerebral ischemic infarction and has good repeatability. The rat MCAO model established by the suture method does not require craniotomy and is less traumatic, thus avoiding damage to the skull structure of the brain (Figure S16, Supplementary Materials). In addition, the MCAO model has the advantages of large infarct volume, high reproducibility, and precise and controllable duration of reperfusion and ischemia. Therefore, the MCAO model is considered to be the most suitable for reproducing the brain injury after ischemic stroke and reperfusion [21]. Additionally, in the test, it was found that the model control group rats had a mortality rate of 25%.

Twenty-four hours after MCAO modeling, the rats were scored for neurological deficits; dead rats were excluded from the experiment, and the neurological function score of 1 to 3 points was selected for the experiment. The rats were tested: the higher the neurological score, the more serious the behavioral disorder of the rats. The neurological deficit scores after administration of each group in the experiment are shown in Table S8 (Supplementary Materials) below. The neurological function scores of each group are as follows: the score of the sham operation group was 0 ± 0.00, the score of the model group was 2.50 ± 0.50, the score of the NBP group was 1.20 ± 0.60, the score of NE group was 1.60 ± 0.92, and the score of NE-ISG group was 1.30 ± 0.78. The results of the study showed that the rats in the sham-operated group did not have any symptoms of neurological loss. Rats in the model group, NE group, NE-ISG group and NBP group were successfully modeled, and they all developed symptoms of neurological deficits, which were manifested as paralysis of the left forelimb, turning to the left or tilting to the left when walking, turning to the left when lifting, etc. After continuous administration for 7 days in each group, the results of neurological deficit score showed that both the Chaxiong volatile oil nanoemulsion group and the Chaxiong volatile oil nanoemulsion in situ gel group had a certain improvement effect on behavioral disorders caused by cerebral ischemia in rats, and the improvement effect after NBP administration is equal. Chaxiong volatile oil nanoemulsion also has a certain improvement effect on neurobehavioral disorders caused by cerebral ischemia in rats.

#### 2.7.2. TTC Staining

After the rat brain tissue was stained with TTC, the normal brain tissue was red, and the cerebral infarction was white. The percentage of cerebral infarction area in each experimental group was measured by Image J software (Wayne Rasband and contributors National Institutes of Health, Bethesda, MD, USA): 0 ± 0.00 in the sham operation group, 41.36 ± 4.83% in the model group, 23.02 ± 2.30% in the NBP group, 31.65 ± 2.60% in the NE group, and 25.14 ± 4.26% in the NE-ISG group.

**Figure 6:**
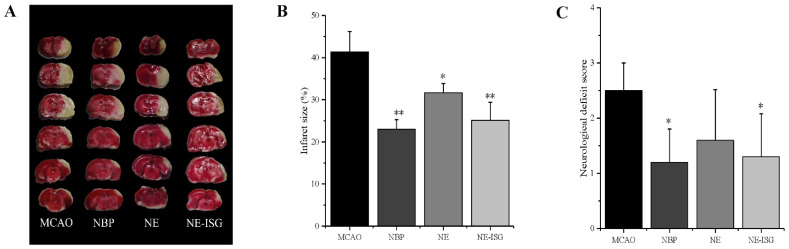
VOC-NE-ISG reduced brain damage and improved functional outcome after cerebral IR (*n* = 10, X ± SD). (**A**) Representative TTC stained serial coronal brain sections; (**B**) mean infarct volumes were detected; (**C**) neurological score. * *p* < 0.05, ** *p* < 0.01.

The measurement results of the cerebral infarction area are shown in Figure 6A and Table S8 (Supplementary Materials). The brain tissue of the rats in the sham operation group was red, and no infarction was found; the brain tissue of the rats in the model group and each drug group appeared as white infarct. From the staining results of brain tissue in Figure 6A, it can be seen that compared with the model group, the infarct area of the rats in the NE-ISG group was significantly reduced (*p* < 0.01), indicating that the volatile oil nanoemulsion in situ gel preparation of Chaxiong obviously improved the effect of cerebral ischemia. Additionally, there was a significant difference between the NE group and the model group (*p* < 0.05), indicating that after intranasal administration of Chaxiong volatile oil nanoemulsion, the area of cerebral infarction in MCAO rats was reduced, and it had an improvement effect on cerebral infarction. After intranasal administration of NBP, the area of cerebral infarction in rats was reduced, and there was a significant difference compared with the model group (*p* < 0.01). The results of cerebral infarction size determination are as follows: NBP group > NE-ISG group > NE group. NBP is a clinical drug for the treatment of ischemic stroke in China, and the effect is significant. Nasal administration of VOC-NE-ISG can achieve drug delivery from nose to brain, increase the transport of drugs to brain tissue, and improve the efficiency of ischemic stroke treatment. After statistical analysis, the NE-ISG group was compared with the model group, *p* < 0.01; compared with the model group, the NE group was *p* < 0.05, indicating a significant difference, indicating that the cerebral infarction area of the rats was improved, and the efficacy of the VOC-NE-ISG group was better than that of the NE group.

## 3. Materials and Methods

### 3.1. Materials

Cremophor RH40 was purchased from Dalian Meilun Biotechnology Co., Ltd. (Dalian, China). A food grade non-ionic surfactant (Tween 80) was purchased from Sichuan Jianshan pharmaceutical Co., Ltd. (Sichuan, China). Span 80 was purchased from Tianjin Damao Chemical Reagent Factory (Tianjin, China). Ultra-MilliQ water was used in the preparation of all solutions and emulsions. Dialysis membrane (MWCO 3.5 kDa) was purchased from Nanjing Senbega Biotechnology Co., Ltd. (Nanjing, China). All other chemicals were of the analytical grade and used as received.

### 3.2. Animals

In vivo studies were conducted using male Sprague Dawley rats weighing 220~250 g. The experimental animals were purchased from Silaikejingda Laboratory animals Co., Ltd., Hunan, China. The animal quality license number was SCXK 2019-0004. This experiment was approved by the Animal Ethics Committee of Jiangxi University of Traditional Chinese Medicine (jzllsc0137). All procedures were conducted in accordance with the ethical guidelines for laboratory animals in China. All efforts were made to minimize both animal suffering and the number of animals used to produce reliable data.

### 3.3. Preparation of Chaxiong Volatile Oil

A measure of 10 kg of dried rhizomes of Chaxiong was crushed, passed through a 20-mesh sieve, and extracted twice with 6 times the amount of 95% ethanol for 1.5 h each time. The filtrate was merged and concentrated under reduced pressure to obtain a concentrated extract, which was dispersed in 2 L water, thoroughly mixed, and extracted with ethyl acetate, and the solvent was recovered under reduced pressure to obtain an ethyl acetate extraction part. Finally, the extraction part was rectified by POPE molecule, and light components (CX-QZF) and heavy components (CX-ZZF) can be obtained at 0.93%.

### 3.4. HPLC Analysis of VOC

The quantification of five phthalides of VOC in formulation samples was determined by a validated reversed-phase HPLC-UV method (Shimdzu, Japan). The HPLC system consisted of a LC-20AT pump, an SPD-M20A PAD-visible detector, a SIL-20A injector with a 20 μL loop and an LC solution workstation. The mobile phase consisted of a mixture of ultrapure water (A) and acetonitrile (B) (0~10 min, 38~42% B; 10~36 min, 42~45% B; 36~55 min, 45~48% B; 55~60 min, 48~38% B) was delivered with a flow rate of 1 mL/min, and separation was performed using an Akzo Nobel Kromasil 100-5-C18 column (4.6 mm × 250 mm, 5 µm) with a sample injection volume of 20 µL. The analysis was performed at 30 °C with UV detection at 280 nm for senkyunolide A(SA) and Z-ligustilide (ZL), and 230 nm for neocnnolide (NOL), n-butylphthalide (NBP) and butenylphthalide (BP) [22].

### 3.5. Screening of Nanoemulsion Formulations

#### 3.5.1. Screening of Oil Phase

The selection of excipient oil (VOC), surfactant and co-surfactant was performed purely on the basis of drug solubility and developed nanoemulsion stability. It was found through preliminary experimental research that the volatile oil of Chaxiong could form a uniform, transparent and stable nanoemulsion. Therefore, the volatile oil of Chaxiong was used as the oil phase of the nanoemulsion in this experiment.

#### 3.5.2. Screening of Cosurfactant

The solubility of volatile oil of Chaxiong in cosurfactant solutions was determined by shake flask method [23]. For cosurfactants, PEG400, glycerin, absolute ethanol, 1,2-propanediol, glycerol, and n-butanol were selected. In brief, 1 g of Chaxiong volatiles was added to a centrifuge tube containing 2 mL of solvent (cosurfactant) and vortexed with the help of a Cyclomixer. Then, the centrifuge tube was oscillated in a gas bath constant-temperature shaker at 37 ± 1.0 °C at 100 r/min for 48 h. Following 48 h, the sample was centrifuged at 3000 rpm for 15 min, and then 100 μL of the supernatant was taken from each co-emulsifier layer, and the volume was made up to 10 mL with methanol and sonicated for 10 min before filtration using nylon filter (0.22 µm). The filtrate was measured at 280 nm and 230 nm by HPLC to estimate the amount of dissolved drug. All measurements were repeated three times.

#### 3.5.3. Screening of Surfactant

For reference methods, different hydrophilic and oleophilic surfactants were used to prepare emulsifiers in a certain proportion (Table S1, Supplementary Materials). The HLB number value of mixed surfactants (A/hydrophilic and B/lipophilic surfactants) was calculated by the following formula: [24]

HLB_mix_ = HLB_A_ × 0.01 W_A_ + HLB_B_ × 0.01 W_B_ (1)

W_A_ + W_B_ = 100%

where HLB_mix_ is the HLB value of the composite emulsifier, HLB_A_ and HLB_B_ were the HLB values of the surfactants A and B, and W_A_ and W_B_ are the mass fractions of the surfactants A and B.

Screening of surfactant was carried out according to the test methods of the literature; Span 80, Tween 80, Span 80 and Tween 80 were compounded in a certain proportion (HLB = 9, 10.5, 12, 13.5) of composite emulsifier, with polyoxyethylene hydrogenated castor oil (RH40) as an emulsifier. Seven 10 mL graduated test tubes with stoppers were randomly selected and numbered, 0.1 g of volatile oil and 0.2 g of co-surfactant were added to each test tube, and then 0.4 g of emulsifier was added to each test tube, and the mixture was mixed with a vortexer. After homogenization, the volume was diluted to 3 mL with ultrapure water, shaken again for 10 min, then the emulsion state and layered volume were observed, and an appropriate amount was taken to dilute 100 times, the solution state was observed, and the particle size was measured.

#### 3.5.4. Pseudo-Ternary Phase Diagrams Construction

An aqueous titration method was utilized to prepare a VOC-NE. Based on solubility studies, the pseudo-ternary phase diagram was constructed with volatile oil of Chaxiong (VOC) as the oil phase, RH40 as the surfactant, anhydrous ethanol as the surfactant, and Mili-Q water as the aqueous phase. Surfactant and cosurfactant are mixed at a fixed weight ratio (K_m_, 1:1, 2:1, 3:1, 4:1, 5:1, *w*/*w*) as a mixed emulsifier (Smix). The mixed emulsifier (Smix) was then mixed with Chaxiong essential oil. For each phase diagram, the ratio of oil to Smix was 9:1, 8:2, 7:3, 6:4, 5:5, 4:6, 3:7, 2:8, and 1:9 (*w*/*w*) [12]. At room temperature, the oil phase and the mixed emulsifier were stirred and mixed uniformly, and the water phase was added dropwise while stirring, until the solution changed from clear to turbid and then clear, or from the turbidity to the critical point of clear, then the amount of water added dropwise was observed and recorded. A pseudo-ternary phase diagram was constructed using Origin software, and the largest Km in the nanoemulsion region was selected as the optimal Km. The physical state of the nanoemulsion was labeled on a pseudo-tricomponent phase diagram with one diagonal of triangle representing the aqueous phase, the other one representing the oily phase, and the third representing a mixture of surfactant and cosurfactants (Smix ratio). 

#### 3.5.5. Optimization of VOC-Nanoemulsion

The Box–Behnken response surface design (BBD) was used to optimize VOC-NE formula parameters by Design-Expert software. According to the results obtained from the preliminary experiment, the ratio of oil (A), the ratio of Smix (B), and the stirring time (C) were selected as the independent variables. According to the experimental design, VOC-NE preparations were optimized by the results of the particle size (Y1), PDI (Y2), and Zeta potential (ZP) (Y3) of the nanoemulsion (Table S2, Supplementary Materials).

Linear, two-factor interaction, and quadratic models (model fit between) were used to predict the optimized formula. Polynomial equations were fitted to each response value from ANOVA and used to assess the effect of the independent variables on the response under study. Models with a *p*-value of less than 0.05 were considered statistically significant. The objective function of this study is to minimize the particle size, PDI, and zeta potential.

#### 3.5.6. Preparation of Volatile Oil of Chaxiong Nanoemulsion

Nanoemulsions were prepared using the emulsion phase inversion (EPI) method [25]. Initially, volatile oil of Chaxiong was used as the oil phase. It was slowly added into a Smix, stirred at fixed speed using a magnetic stirrer at room temperature. Additionally, the aqueous phase was slowly added to the organic phase under agitation and continually stirred for a certain time to obtain the volatile oil of Chaxiong nanoemulsion (VOC-NE). 

### 3.6. In Vitro Evaluation of Volatile Oil of Chaxiong Nanoemulsion

#### 3.6.1. Traits and Types

The properties of the prepared VOC-NE were observed by the naked eye. Identification of VOC-NE by dyeing method was oil-in-water (O/W) or water-in-oil (W/O) nanoemulsion; identification of the system properties of the volatile oil of Chaxiong nanoemulsion was achieved by conductivity method.

#### 3.6.2. Particle Size, Polydispersity Index and Zeta Potential

The particle size, polydispersity index (PDI) and zeta potential of Chaxiong volatile oil nanoemulsion were determined by photon correlation spectroscopy using a Malvern Zetasizer (Nano ZS 90, Malvern Instruments, Malvern City, UK) [26]. The zeta potential was a very useful method to assess the stability of any colloidal system. The prepared VOC nanoemulsion was diluted 200 times with ultrapure water, equilibrated at 25 °C for 120 s, and the measurement was repeated three times for each sample to obtain the average particle size (mean ± SD) of the nanoemulsion. Before measurement, the sample was properly diluted with ultrapure water to avoid the phenomenon of multiple scattering. Equilibration was performed at 25 °C, the angle of analysis was 173°, and each sample was measured in triplicate to obtain the mean zeta potential value of the nanoemulsion.

#### 3.6.3. pH, Viscosity, Refractive Index and Transmittance

The pH of the VOC-NE formulation was determined by using a calibrated digital pH meter (METTLER TOLEDO) at room temperature. The pH meter was calibrated with calibration solutions of different pH before use, and the pH values of all the Chaxiong volatile oil nanoemulsion preparations were determined in triplicate. The viscosity of VOC-NE was measured in triplicate using a rotational viscometer (NDJ-9S, Shanghai, China) at room temperature without dilution. The refractive index (RI) of the prepared nanoemulsions was measured at room temperature using an Abbe refractometer normalized with castor oil. A few drops of formulation were placed on glass slides and the RI was determined in triplicate. In order to obtain an idea regarding the stability of the nanoformulation, the percentage transmittance for the developed formulations was measured by UV–Vis spectrophotometer (UV-2550, Shimadzu, Kyoto, Japan) at 650 nm. The blank used was MilliQ water, whereas no dilution for any formulation was carried out in this process.

$$A=\log\left(\frac{1}{T}\right)$$

#### 3.6.4. Transmission Electron Microscopy (TEM)

The appearance and morphology of Chaxiong volatile oil nanoemulsion were studied by transmission electron microscopy (JEM-2100, Shimadzu, Japan) [27]. After a 5-fold dilution, the formulations were placed on a 400-mesh copper net coated with a carbon film and dried for 3–5 min at room temperature, the excess liquid was absorbed with filter paper, negatively stained with 2% phosphotungstic acid for 30 s, and placed for 2–3 min to dry naturally, then the morphology of nanoemulsion was observed under a transmission electron microscope. The morphology of nanoemulsions can be observed by scanning with a transmission electron microscope.

#### 3.6.5. Stability Studies

The physical stability of the selected nanoemulsions was studied. Firstly, 2 mL of VOC-NE formulation was measured into a centrifuge tube and the samples were centrifuged at 3500 rpm for 15 min [28]. The freeze–thaw stability of VOC-NE formulation was studied by three freeze–thaw cycles between −20 °C and +25 °C, and the preparation was placed at each temperature for 48 h. The NE formulations were subjected to six heating–cooling cycles at 4 °C and 45 °C for at least 48 h at each temperature [29]. The formulations were observed for physical stability, globule size and ZP before and after each method. The NEs that passed the stability tests were selected for the formulation of in situ gels.

### 3.7. Screening of VOC-NE-ISG Gel Framework Materials

#### 3.7.1. The Effect of P407 on the Gelling Temperature of In Situ Gels

The effects of different concentrations of poloxamer 407 solutions on the gelation temperature were investigated. Taking five 50 mL beakers, P407 was added in proportions of 16%, 18%, 20%, 22%, and 24% (*w*/*w*), respectively, then a certain amount of 4 °C ultrapure water was added, and a total of 10 g A series of poloxamer solutions of different concentrations was prepared. The gelation temperature (°C) of each poloxamer solution was determined by the test tube inversion method, each sample was replicated in 3 replicates, and the average value was taken.

#### 3.7.2. The Effect of P188 on the Gelation Temperature of In Situ Gels

The effects of different concentrations of poloxamer 188 solutions on the gelation temperature were investigated. Taking five 50 mL beakers, a certain proportion of P407 was added, then P188 was added in a proportion of 2%, 4%, 6%, 8%, and 10% (*w*/*w*), and a certain amount of 4 °C ultrapure water was added. A series of mixed poloxamer solutions of different concentrations were formulated in a total amount of 10 g. The gelling temperature (°C) of each mixed poloxamer solution was determined by the test tube inversion method, and each sample was replicated in 3 replicates, and the average value was taken.

#### 3.7.3. Screening Prescription Ratio of VOC-NE-ISG

The proportion of P407 was fixed at 10%, 12%, and 14%; the proportion of P188 was added at 2%, 4%, 6%, 8%, and 10%, respectively; and the proportion of prescriptions of VOC-NE-ISG was screened.

#### 3.7.4. Preparation of VOC-NE-ISG

The volatile oil of chaxiong nanoemulsion in situ gel (VOC-NE-ISG) was prepared by an improved cold method [30]. The gel bases P407 and P188 were weighed according to the prescription ratio, then they were put in a beaker, slowly adding a certain amount of volatile oil of Chaxiong nanoemulsion under continuous stirring, and stirred to make it evenly dispersed, then stored at 4 °C and mixed regularly until the poloxamer swelled completely to obtain a clear, uniform solution without clumps.

### 3.8. In Vitro Evaluation of VOC-NE-ISG

#### 3.8.1. Measurement of pH and Viscosity of the Gel

The pH of VOC-NE-ISG formulation was determined using a pH meter (METTLER TOLEDO) at room temperature. The viscosity of VOC-NE-ISG samples was measured by a rotational viscometer (NDJ-9S, Shanghai, China). An appropriate amount of VOC-NE-ISG formulation was put in a 250 mL beaker, rotated at 12 r/min with a No. 4 rotor, and the viscosity of the sample at room temperature and gelation temperature was measured. The pH and viscosity of the developed VOC-NE-ISG was determined in triplicate. 

#### 3.8.2. Measurement of Gelation Temperature and Gelation Time

The gelation temperature and gelation time of VOC-NE-ISG formulation was determined by the test tube inverting method [31]. A measure of 2 mL of formulation was added to a 10 mL glass test tube and kept in a water bath. The initial temperature of the water bath was set at 20 °C, and the equilibration time was 10 min. The rotor was added to ensure uniform temperature diffusion, controlling the heating rate at 0.5~1.0 °C/min. The temperature was raised from 20 °C to 40 °C. The test tube was removed and inverted every 1 min to observe the state of the sample. The gel point was determined by an inverted tube in 30 s with or without flow criteria. The temperature for gelation was noted in triplicate.

A measure of 2 mL of VOC-NE-ISG formulation was added to a 10 mL test tube, equilibrated at a constant-temperature water bath at 20 °C for 10 min, then placed in a water bath at 33 °C, then the timing was started immediately. The gelation time is the time required for the VOC-NE-ISG sample to change from a semi-solid gel-like substance to the gel solution no longer flowing within 5 s.

#### 3.8.3. Measurement of Gel Strength

A 50 g sample of VOC-NE-ISG was placed in a 100 mL cylinder and gelated in a thermostatic water bath at 32–34 °C. Then, a 35 g weight was placed on a disc with a diameter of 2.3 cm, a gap of 0.4 cm in the side walls of the cylinder, and a thickness of 0.5 cm, and the disc was placed on the gel, as shown in Figure S1. The gel strength is the time (s) required to move the piston down 5 cm in the gel. In cases in which it takes more than 5 min to put the device into the gel without dropping to 5 cm, an additional weight is placed on top of the device, and the minimum weight to push the device down 5 cm into the gel is the gelation strength of the gel [32].

#### 3.8.4. Measurement of Bioadhesion

Bioadhesion is the force required to detach an in situ gel formulation from nasal mucosal tissue. The mucoadhesion of in situ gel formulations was determined using a modified mucoadhesion measurement device [33]. The modified equilibration technique used two glass vials and porcine nasal mucosa. The thickness of the nasal mucosa is approximately 0.6 mm, and the mucosal side is separately fixed to each glass vial. The glass vial was placed at 32–34 °C for 10 min to keep its overall temperature around 33 °C to prevent hydration of the gel during the measurement. One vial was attached to one side of the scale, the other vial was placed directly below it, and a 0.5 mL gel sample was placed between the two mucous membranes attached to the bottom of the vial. The minimum weight required to break the mucoadhesion, which is m, was measured. The gel bioadhesion measurement device is shown in Figure S2 (Supplementary Materials).

(2) $$f\left(\mathrm{dynes}/{\mathrm{cm}}^{2}\right)=\mathrm{mg}/A^{2}$$

where m is the weight (g) required to separate the mucosa, g is the acceleration due to gravity (980 cm/s^2^) and A is the mucosal surface area (cm^2^) of the formulation in contact with the mucosa.

#### 3.8.5. Measurement of Expansion Coefficient and Gel Water Retention Capacity

The volume of the in situ gel formed after the solution-gel phase transition is larger than that of the solution. When the gel swells, it may block the nasal passages and cause discomfort to the patient. Therefore, in order to avoid discomfort caused by gel swelling after intranasal administration, an in situ gel swelling study was performed [34]. Briefly, 2 mL of VOC-NE-ISG was placed in a test tube with a stopper and scaled and placed in a 25 °C water bath, and the volume V_0_ of the in situ gel at the moment was recorded. The centrifuge tube was placed in a 33 °C water bath and the transformed gel volume V_G_ was recorded. The formula for the expansion coefficient is as follows:

(3) $$S\%=\frac{V_{G}-V_{0}}{V_{0}}\times 100$$

The water-holding capacity of the VOC-NE-ISG was evaluated using the method described by Hosny K M et al. [35]. A measure of 1 mL of Chaxiong volatile oil nanoemulsion in situ gel solution was placed in a precisely weighed centrifuge tube, 0.25 mL of SNF was added, and the solution was mixed evenly to form a gel-like ISG, and the gel mass was recorded as M_1_. After it was centrifuged at 6000 rpm for 15 min, the separated water layer was sucked off with filter paper, and the gel mass at this time was recorded as M_2_. The test was carried out at 33 °C, and the test was repeated 3 times. Preparation of simulated nasal fluid (SNF): Accurately weigh 8.77 g of NaCl, 2.98 g of KCl, and 0.45 g of CaCl_2_ 2H_2_O, then add an appropriate amount of ultrapure water to dissolve and dilute to 1000 mL.

(4) $$\mathrm{Gel}\;\mathrm{water}\;\mathrm{retention}\;\mathrm{capacity}\left(\%\right)=\frac{M_{2}}{M_{1}}\times 100$$

#### 3.8.6. Measurement of Rheological Properties

The rheological behavior of VVOC-NE-ISG was studied by a modern rheometer MCR101 (Anton Paar Inc., Graz, Austria). The sample (1.2 g) was placed in a beaker and equilibrate for 1 min before measuring. Firstly, amplitude scanning was performed on the VOC-NE-ISG to determine the linear viscoelastic region of the sample. Next, the elastic modulus (G′) and loss modulus (G″) of the VOC-NE-ISG formulation were measured at 37 °C with a shear rate of 0–100 s^−1^ [36]. Additionally, through amplitude scanning and frequency scanning, the changes in G′, G″, angular frequency and strain of the sample were studied. The viscosity of the in situ gel was measured at a constant shear rate of 100 s^−1^ using a rheometer at 34 °C. 

#### 3.8.7. Measurement of Percent Drug Content

For determination of the drug content, about 1 mL of VOC-NE-ISG was taken and diluted appropriately with methanol up to 25 mL and analyzed by HPLC at 280 and 230 nm. The drug content determination was made in triplicate.

Five components in the volatile oil of Chaxiong were analyzed by HPLC. The results of the methodological study showed that the method had good specificity, linearity, sensitivity, accuracy, and precision. The results of the linear relationship showed that the concentration range of ZDJBT, YCXNZ, XSCNZ, GBNZ and DXJBT were 0.19–19.44 µg/mL, 0.30–300.03 µg/mL, 0.26–130.26 µg/mL, 0.53–267.07 µg/mL and 0.23~22.61 µg/mL, respectively. Additionally, R^2^ is greater than or equal to 0.9998 in their respective linear ranges. The limit of detection of ZDJBT, YCXNZ, XSCNZ, GBNZ, and DXJBT was 194.40, 260.52, 300.03, 534.14, and 226.10 ng/mL, respectively. 

#### 3.8.8. Measurement of In Vitro Release Studies 

In vitro release studies were carried out by using the dialysis method [37]. Dialysis bags with a molecular weight cutoff of 3.5 kDa were immersed in the release medium for 24 h before the experiment. The VOC-NE and VOC-NE-ISG formulations were inserted into the semipermeable dialysis bag. Additionally, both sides of the dialysis bag were sealed with a thread to prevent leakage. The dissolution tester (Vision classic 6, Hanson, America) was used for the release test. A measure of 900 mL of phosphate-buffered solution (PBS) (pH 6.4) was used as the release medium. The release temperature was kept at 37 °C ± 0.5 °C, and 50 rpm was stirred.

At a predetermined timepoint, 1 mL of the sample was taken out and replaced with the same volume of fresh PBS. The withdrawn samples were analyzed for the concentration of five phthalides of the VOC formulation using the HPLC method previously described after filtration through a membrane (φ = 0.45 μm). All experiments were performed in triplicate.

The release kinetic of five components of VOC from the formulations was studied by fitting different kinetic models such as the zero-order, first-order, Higuchi, and Ritger–Peppas models.

#### 3.8.9. Histological Evaluation of Nasal Mucosa

The irritation of VOC-NE-ISG to nasal mucosa was investigated by pathological sections of nasal mucosa [20]. Twelve male SD rats with a body weight of 220–250 g were selected and randomly divided into 3 groups with 4 rats in each group. A negative control group, positive control group, and experimental group, respectively, were given nasal saline, VOC-NE-ISG solution, and isopropanol solution. A measure of 50 μL of the drug solution was injected into the left and right nostrils of the rats in each group with a micro-syringe, and the drug was administered continuously for 7 days, once a day. After 24 h of administration, on the 7th day, the rats were anesthetized by intraperitoneal injection of 10% chloral hydrate solution and sacrificed by decapitation. The nasal mucosa was removed to observe whether there were congestion, edema, and necrosis. Histopathological sections of nasal mucosa were performed by the biological tissue embedding section method, which were dewaxed and stained, mounted, and observed under an optical microscope.

### 3.9. Pharmacokinetic Studies

#### 3.9.1. Animal Studies

Thirty male Sprague Dawley rats weighing 240 ± 10 g were used for this study. They were randomly divided into five groups (VOC intravenous injection group, VOC gavage group, VOC nasal solution group, VOC-NE group and VOC-NE-ISG group), fasted for 24 h prior to drug administration but allowed free access to water. The volatile oil of Chaxiong intravenous injection and oral solution were prepared by dissolving 192.0 mg of Chaxiong volatile oil in 25 mL of normal saline containing 0.5 g Tween-80 as a solubilizer. The solution for nasal administration of the volatile oil of Chaxiong was obtained by dissolving 664.4 mg volatile oil of Chaxiong in 10 mL ultrapure water added with Tween 80 as a solubilizer.

Before the test, the rats were weighed and injected intraperitoneally with 10% chloral hydrate solution. After the rats were anesthetized, five different preparations of Chaxiong volatile oil were given, respectively, through the tail vein, gavage, and nasal cavity. The first group was administered by tail vein injection of Chaxiong volatile oil solution; the dose was 15 mg/kg. In the second group, the volatile oil solution of Chaxiong was administered by gavage; the dose was 30 mg/kg. In the third group, the nasal administration group of Chaxiong volatile oil solution, the dose was 13.29 mg/kg, and the administration volume was 50 μL/rat. In the fourth group, the intranasal administration group of Chaxiong volatile oil nanoemulsion, the dose was 13.32 mg/kg, and the administration volume was 50 μL/rat. In the fifth group, the intranasal administration group of Chaxiong volatile oil nanoemulsion in situ gel, the dose was 10.4 mg/kg, and the administration volume was 100 μL/rat.

For nasal administration, a 50 µL microsyringe was used, and a tube with a diameter of about 1 mm was connected to the needle to absorb the drug solution, and the tube was inserted into the rat nostril about 5–10 mm to push the drug solution. A measure of 0.5 mL of blood was collected from the orbital venous plexus at 5, 15, 30, 45, 60, 90, 120, 240, and 360 min after administration, respectively. The blood samples were placed in a 1.5 mL EDTA anticoagulant tube, centrifuged, and the supernatant was separated and stored at −80°C. The plasma samples were processed and then injected for measurement, and the concentration of Senkyunolide A, n-butylphthalide, neocnidolide, Z-ligustilide and butenylbenzene in rat plasma was calculated. 

#### 3.9.2. Blood Sample Analysis

A measure of 200 μL of rat plasma sample supernatant was precisely pipetted and placed in a 1.5 mL centrifuge tube. Then, 20 μL internal standard working solution was added, followed by 50 μL 2% formic acid and 400 μL methanol. The mixture was vortex mixed for 2 min and centrifuged at 13,000 r/min for 15 min at 4 °C. The supernatant was taken, dried by nitrogen flow at 30 °C, redissolved by adding 100 μL methanol, and centrifuged at 13,000 r/min for 15 min after vortexing. The supernatant was absorbed and injected into the sample for determination.

#### 3.9.3. UPLC-MS/MS Method

An HPLC system consisting of a LC-30 AD solvent-delivery system, an SIL-30 A Cautosampler, a CTO-30 AC column oven, a DGU-20A3 solvent degasser, and a CBM-20A controller of AB Sciex (Framingham, MA, USA) were used in the study. The Shim pack GIST C18-AQ high-pressure chromatographic column (2.1 mm × 100 mm, 3 μm) was performed for component separation. The mobile phase was 0.1% formic acid aqueous solution (A) and acetonitrile (B), and the gradient elution procedure was 0–2 min, 45–60% B; 2–6 min, 60–65% B; 6–7 min, 65–95% B; 7–9 min, 95- 45% B and 9–10 min, 45% B, with the flow rate maintained at 0.3 mL/min. The injection volume was set at 2 μL and the column oven was maintained at 35 °C [22]. 

AB Science is equipped with Turbo V source and Turbo ion spray™ 4500 QTRAP interface™ (AB SCIEX, Framingham, MA, USA), which was used for mass spectrometry.

Electrospray ionization was performed in positive mode. The detection method was multiple reaction monitoring (MRM) with an ion source temperature of 500 °C and a desolvent gas temperature of 550 °C. The mass spectrum conditions of each index component and internal standard are shown in Table S3 (Supplementary Materials).

### 3.10. In Vivo Brain Targeting Studies

#### 3.10.1. Animal Studies

To investigate the brain distribution of VOC, 105 male Sprague Dawley rats were randomly divided into five groups for brain tissue sample collection after administration. Before the test, the rats were weighed and injected intraperitoneally with 10% chloral hydrate solution. After the rats were anesthetized, three kinds of volatile oil of Chaxiong preparations were given, respectively, through the tail vein, gavage, and nasal cavity. The grouping and administration of each group are as described in the previous “3.9.1”.

For nasal administration, a 100 µL microsyringe was used, and a tube with a diameter of about 1 mm was connected to the needle to absorb the drug solution, and the tube was inserted into the rat nostril about 5–10 mm to push the drug solution. At 15, 30, 60, 90, 120, 240, and 360 min after administration, 0.5 mL of blood was collected from the orbital venous plexus (*n* = 3 at each time point), centrifuged at 8000 r/min for 10 min, and the supernatant was separated, then stored at −80 °C in a refrigerator. Immediately after blood collection, the rats were sacrificed by decapitation of the cervical spine, the rats were decapitated, and the brain tissue was quickly separated. Each rat’s brain tissue was quickly rinsed with normal saline to remove blood stains, blotted dry with filter paper, and weighed. After weighing, normal saline was added at a ratio of 1:2 (*w*/*v*), and a tissue homogenizer was used to homogenize to homogeneity. All plasma samples and brain homogenates were stored in a −80 °C freezer until HPLC analysis.

The brain targeting index of VOC-NE-ISG formulation was calculated by the brain targeting index formula to evaluate the brain targeting effect of Chaxiong volatile oil nanoemulsion in situ gel intranasal administration [33].

(5) $$\mathrm{Brain}\;\mathrm{Targeting}\;\mathrm{Index}\;(\mathrm{BTI})=\frac{{\left({\mathrm{AUC}}_{\mathrm{brain}}/{\mathrm{AUC}}_{\mathrm{blood}}\right)}_{\mathrm{in}}}{{\left({\mathrm{AUC}}_{\mathrm{brain}}/{\mathrm{AUC}}_{\mathrm{blood}}\right)}_{\mathrm{ig}}}$$

where AUC_brain_ and AUC_blood_ represent the area under the drug concentration–time curve of each component in brain tissue and plasma, respectively; in represents intranasal administration; ig represents intragastric administration.

#### 3.10.2. Analytical Procedures

The brain tissue analysis of the five components of the volatile oil of Chaxiong was carried out using the reported UPLC-MS analysis method [22]. Rat brain tissue was added to normal saline at a ratio of 1:2 (*w*/*v*), homogenized to obtain a homogenate, and the homogenate was centrifuged at 13,000 r/min for 10 min to separate the brain tissue homogenate supernatant. A measure of 200 μL of the supernatant was precisely pipetted into an EP tube, 20 μL of the internal standard working solution was added, and then 200 μL of n-hexane solution was added, followed by vortex extraction for 3 min and centrifugation at 13,000 r/min for 10 min at 4 °C, then the supernatant was taken for UPLC-MS/MS injection analysis. The UPLC-MS/MS method was as described in “3.9.3” above. Data were analyzed using DAS software version 3.0 (Beijing JiDaoChengran Technology Co., Ltd, Beijing, China).

### 3.11. Pharmacodynamic Studies

#### 3.11.1. Establishment of a Rat Model of Middle Cerebral Artery Occlusion

Middle cerebral artery occlusion (MACO) is a common ischemia-reperfusion model. The suture causes the blood supply of the middle cerebral artery blood supply area to cause focal blood supply to the brain. The cerebral ischemia-reperfusion model was prepared by the suture method [38]. Before the operation, the experimental rats were fasted for 12 h. The rats were anesthetized by intraperitoneal injection of 10% chloral hydrate solution. The anesthetized rats were fixed in a supine position, the hairs of the necks were removed, and they were disinfected with iodophor, opened along the midline of the neck, and the fascia was bluntly separated to expose the rat’s common carotid artery; the “Y”-shaped bifurcation was found, the common carotid artery (CCA), internal carotid artery (ICA), and external carotid artery (ECA) were separated, and the proximal CCA and distal ECA were ligated with 5–0 wire. The ICA was temporarily blocked, and a small opening was made in the common cervical pulse 4 mm from the bifurcation using ophthalmic forceps, and the suture was inserted into the opening of the common carotid artery. Feeling that the suture was blocked, and at the same time, the black mark on the suture was too far from the trifurcation of the common carotid artery, the suture was used to fix the suture, and the wound was sutured. At the same time, the temperature of the animals was maintained at 37 °C with a heating pad. Two hours after the suture was inserted, the suture was gently pulled out of the internal carotid artery and reperfused for 24 h.

#### 3.11.2. Grouping and Administration

The rats after modeling were randomly divided into 5 groups with 10 rats in each group, and the rats were continuously administered for 7 days after the successful modeling of MCAO. The patients were divided into (1) sham operation group (sham group): trauma was caused during the operation, no embolization thread was inserted, and no cerebral embolism ischemia was caused, and 50 uL of normal saline was given to the nasal cavity; (2) model group: the nasal cavity after successful MCAO modeling was administered normal saline; (3) NBP group: 5.4 mg/kg butylphthalide by intragastric administration after successful MCAO modeling (converted according to the human equivalent dose); (4) VOC-NE group: intranasal administration of VOC after successful MCAO modeling -NE 50 µL (13.2 mg/kg calculated by VOC); (5) VOC-NE-ISG group: 100 µL of VOC-NE-ISG (10.4 mg/kg calculated by VOC) was administered nasally after MCAO modeling.

#### 3.11.3. Neurological Deficit Score

Rats were scored after 24 h of cerebral ischemia and reperfusion. In the neurological function score, all experimental rats were treated mildly to avoid causing additional damage to the rats and affecting the final experimental results. Using the Zea-longa 5-point scale, scoring standard: no obvious symptoms of nerve injury, 0 points; if the forepaw could not be fully extended, 1 point; turning to the opposite side while walking, 2 points; walking to the opposite side, 3 points; inability to walk spontaneously and lack of self-awareness, 4 points.

#### 3.11.4. Infarct Volume Evaluation

After reperfusion for 24 h and administration for 7 days, the rats in each group were killed by cervical vertebrae, and the rat brain tissue was quickly taken out, frozen and fixed in a −20 °C refrigerator for 30 min, then the whole brain coronal plane was taken out and cut into 6 slices. The brain tissue was soaked in 2% TTC solution, incubated in a water bath at 37 °C, stained in the dark for 20 min, and finally fixed with 4% paraformaldehyde and photographed [39]. The photos were imported into image software to calculate the cerebral infarct size. The cerebral infarction area was calculated according to the following formula:

(6) Cerebral infarction area=area of white infarct area/total area of the entire brain ×100%

### 3.12. Statistical Analysis

All data were expressed in mean ± SD. SPSS 26.0 software (International Business Machines Corporation, New York, NY, USA) was used for one-way ANOVA and pairwise comparisons with Tukey’s test or LSD test.

## 4. Conclusions

In this study, we propose that the nanoemulsion in situ gel-encapsulated drug is an effective nasal-to-brain drug-delivery system that can enhance the delivery of the essential oil of Chaxiong to the brain for the treatment of ischemic stroke. Pharmacokinetics and brain tissue distribution studies showed that the absorption of volatile oil of Chaxiong into the brain after intranasal administration of VOC-NE-ISG was significantly higher than that of Chaxiong volatile oil solution, which indicated that nanoemulsion modification could be combined by intranasal administration, enhancing drug transport to the brain. It shows that the intranasal administration of Chaxiong volatile oil nanoemulsion gel in situ has obvious targeting and can realize the delivery from the nose to the brain. Pharmacodynamic studies showed that MCAO rats treated with Chaxiong volatile oil nanoemulsion in situ gel showed significantly improved neurological symptoms of MCAO rats, significantly reduced cerebral infarct size, and had a significant anti-ischemic stroke effect. All in all, the results suggested that the drug delivery system in this study could provide an effective noninvasive approach to encourage the access of VOC to the brain for treatment of ischemic stroke.

## Figures and Tables

**Table 1:** Experimental trials, independent variables and observed response for Box–Behnken design used for preparation of VOC-NE. (*n* = 3, X ± SD).

Run	Coded Value			Actual Value			Response Values		
	A	B	C	A/%	B/%	C/h	Size/nm	PDI	ZP/mV
1	1	0	−1	16	25	0.5	70.16 ± 0.88	0.49 ± 0.01	−11.40 ± 1.30
2	0	−1	1	10	20	1.5	33.46 ± 0.35	0.37 ± 0.00	−14.20 ± 1.75
3	0	0	0	10	25	1.0	29.83 ± 0.35	0.34 ± 0.04	−21.77 ± 1.25
4	0	1	−1	10	30	0.5	81.40 ± 0.66	0.57 ± 0.04	−12.30 ± 0.60
5	1	0	1	16	25	1.5	84.95 ± 1.34	0.58 ± 0.02	−14.30 ± 1.00
6	−1	0	1	4	25	1.5	18.02 ± 1.15	0.27 ± 0.02	−12.70 ± 1.82
7	0	0	0	10	25	1.0	24.04 ± 0.92	0.27 ± 0.01	−21.17 ± 0.80
8	0	0	0	10	25	1.0	31.28 ± 1.04	0.44 ± 0.03	−21.95 ± 2.79
9	0	0	0	10	25	1.0	40.47 ± 0.98	0.25 ± 0.03	−22.33 ± 1.19
10	0	−1	−1	10	20	0.5	92.63 ± 0.13	0.69 ± 0.00	−15.90 ± 0.44
11	1	1	0	16	30	1.0	51.37 ± 0.61	0.17 ± 0.01	−14.70 ± 1.79
12	0	0	0	10	25	1.0	26.98 ± 1.56	0.26 ± 0.04	−22.3 ± 0.57
13	−1	1	0	4	30	1.0	43.72 ± 0.32	0.14 ± 0.01	−13.80 ± 1.15
14	0	1	1	10	30	1.5	60.81 ± 0.94	0.35 ± 0.03	−14.59 ± 1.23
15	1	−1	0	16	20	1.0	82.74 ± 0.13	0.41 ± 0.00	13.73 ± 1.15
16	−1	0	−1	4	25	0.5	43.46 ± 0.39	0.20 ± 0.01	−14.10 ± 2.23
17	−1	−1	0	4	20	1.0	19.49 ± 1.14	0.25 ± 0.01	−18.50 ± 0.85

**Table 2:** Table of pharmacokinetic parameters of plasma sample of each index component in each group (*n* = 6, X ± SD).

Active Ingredients	Group	Cmax/μg/L	Tmax/h	t_1/2_/h	MRT (0–t)/h	AUC (0–t)/ μg/L·h	F/%
SA	I.V.	1976.25 ± 1043.97	-	1.63 ± 0.43	1.15 ± 0.24	1191.45 ± 470.03	-
	I.P.	245.40 ± 98.34 *	0.21 ± 0.07	1.75 ± 0.55	1.75 ± 0.15 *	409.66 ± 56.36 *	17.19
	Solution	321.00 ± 21.68 *	0.30 ± 0.11	1.77 ± 0.18	1.59 ± 0.25	284.05 ± 32.17 *	26.91
	NE	349.17 ± 53.24 *	0.40 ± 0.54	2.17 ± 0.38	1.92 ± 0.40 *	334.52 ± 88.13 *	31.94
	NE-ISG	404.50 ± 53.93 *	0.30 ± 0.11	5.72 ± 1.84	1.96 ± 0.13 *	247.34 ± 58.91 *	42.16
NOL	I.V.	1068.80 ± 665.27	-	1.55 ± 0.22	1.21 ± 0.16	477.08 ± 142.35	-
	I.P.	103.19 ± 33.56 *	0.29 ± 0.10	1.93 ± 0.28	1.95 ± 0.33	183.11 ± 47.39 *	19.19
	Solution	132.03 ± 20.05 *	0.40 ± 0.13	2.51 ± 0.27 #	1.73 ± 0.18	107.92 ± 18.82 *	25.53
	NE	114.33 ± 18.97 *	0.50 ± 0.31 *	2.09 ± 0.66	1.70 ± 0.54	119.43 ± 39.78 *	30.62
	NE-ISG	141.83 ± 45.91 *	0.33 ± 0.20	3.14 ± 0.85 #	2.17 ± 0.25	90.08 ± 34.35 *	40.69
NBP	I.V.	97.46 ± 77.23	-	1.58 ± 0.57	1.37 ± 0.69 #	115.65 ± 50.75	-
	I.P.	19.34 ± 8.09 *	0.25 ± 0.16 *	1.88 ± 0.47	2.39 ± 0.21 *	42.38 ± 13.92 *	18.32
	Solution	30.43 ± 6.38 *	0.30 ± 0.11	1.65 ± 0.48	1.75 ± 0.27 #	26.77 ± 17.75 *	26.13
	NE	24.00 ± 6.58 *	0.50 ± 0.27 *	2.74 ± 0.77	2.06 ± 0.85 #	22.59 ± 6.55 *	25.90
	NE-ISG	19.54 ± 2.68 *	0.40 ± 0.14	3.22 ± 0.89	2.27 ± 0.40 #	16.78 ± 1.36 *	34.74
ZL	I.V.	1698.00 ± 904.47	-	1.15 ± 0.81	1.13 ± 0.29	1182.96 ± 155.52	-
	I.P.	232.08 ± 41.33 *	0.23 ± 0.17	1.59 ± 0.77	1.29 ± 0.49	225.77 ± 85.89 *	9.54
	Solution	231.25 ± 15.84 *	0.56 ± 0.13 *	1.59 ± 0.48	1.50 ± 0.25	223.06 ± 14.49 *	21.28
	NE	234.5 ± 64.40 *	0.37 ± 0.25 *	2.81 ± 1.59	1.78 ± 0.27	245.38 ± 37.83 *	26.03
	NE-ISG	225.63 ± 49.14 *	0.50 ± 0.11 *	3.78 ± 2.06	3.48 ± 0.34	207.02 ± 18.32 *	29.93
BP	I.V.	173.30 ± 118.63	-	0.88 ± 0.42	1.16 ± 0.45	144.72 ± 42.33	-
	I.P.	17.70 ± 3.57 *	0.50 ± 0.20	1.79 ± 0.46	1.85 ± 0.49	31.59 ± 4.14 *	10.91
	Solution	20.47 ± 5.93 *	0.23 ± 0.17	1.95 ± 0.61	1.78 ± 0.39	29.74 ± 10.38 *	23.19
	NE	17.00 ± 3.43 *	0.55 ± 0.54	1.98 ± 1.13	2.58 ± 1.03	27.04 ± 8.33 *	26.30
	NE-ISG	17.08 ± 2.20 *	0.50 ± 0.25 *	3.09 ± 0.89 *	2.79 ± 1.25 *	17.63 ± 4.60 *	31.63

Note: * *p* < 0.05 vs. intravenous group, # *p* < 0.05 vs. gavage group.

**Table 3:** Table of pharmacokinetic parameters of plasma and brain tissue of each index component in each group (*n* = 3, X ± SD).

Active Ingredients	Group	Sample	C_max_/μg/L	T_max_/h	t_1/2_/h	MRT_(0–t)_/h	AUC_(0–t)_/μg/L·h	BTI
SA	I.V.	blood	1680.00 ± 164.84	-	1.14 ± 0.68	0.79 ± 0.18	1751.66 ± 98.68	0.22
		brain	74.20 ± 3.81	1.17 ± 0.28	2.08 ± 0.53	1.32 ± 0.19	187.13 ± 26.19	
	I.P.	blood	289.66 ± 47.37 *	0.50 ± 0.00	1.13 ± 0.44	1.12 ± 0.06	347.43 ± 32.39 *	-
		brain	102.00 ± 39.46	0.68 ± 0.28	1.08 ± 0.34	1.53 ± 0.18	166.04 ± 21.07	
	Solution	blood	248.58 ± 77.17 *	0.41 ± 0.14	0.94 ± 0.19	1.46 ± 0.35	304.41 ± 23.07 *	0.97
		brain	123.63 ± 59.65	0.83 ± 0.28	1.70 ± 1.07	1.96 ± 0.43	140.48 ± 55.01	
	NE	blood	200.67 ± 39.95 *	0.66 ± 0.28	1.84 ± 0.50 *	1.93 ± 0.51 *	350.60 ± 28.64 *	1.19
		brain	127.00 ± 31.11	0.58 ± 0.38	2.95 ± 1.07	2.31 ± 0.44 *	199.74 ± 31.20	
	NE-ISG	blood	377.66 ± 196.13 *	0.50 ± 0.00	1.04 ± 0.63	2.09 ± 0.72 *	273.5 ± 18.30 *	1.73
		brain	236.00 ± 28.28 * #	0.50 ± 0.14	5.17 ± 4.25	2.44 ± 0.19 *	226.65 ± 1.91	
NOL	I.V.	blood	863.33 ± 122.91	-	1.23 ± 0.71	1.15 ± 0.22	1138.52 ± 58.62	0.22
		brain	70.76 ± 20.99	0.83 ± 0.28	1.77 ± 1.29	1.61 ± 0.22	118.20 ± 23.39	
	I.P.	blood	187.83 ± 69.37 *	0.41 ± 0.14	0.79 ± 0.07	1.05 ± 0.19	188.40 ± 27.95 *	-
		brain	56.50 ± 16.26	0.66 ± 0.28	0.97 ± 0.34	1.47 ± 0.78	90.85 ± 526.80	
	Solution	blood	192.33 ± 69.24 *	0.66 ± 0.28	1.58 ± 0.92	1.27 ± 0.42	246.56 ± 4.98 *	0.83
		brain	67.16 ± 11.18	0.33 ± 0.14	1.61 ± 1.90	1.39 ± 0.75	98.19 ± 72.38	
	NE	blood	236.35 ± 48.38 *	0.66 ± 0.28	1.73 ± 0.28	1.82 ± 0.40	250.80 ± 90.78 *	1.14
		brain	73.33 ± 46.07	0.50 ± 0.66	2.62 ± 1.54	2.34 ± 0.34	138.23 ± 60.64	
	NE-ISG	blood	216.00 ± 77.78 *	0.50 ± 0.00	2.57 ± 0.52	1.71 ± 0.39	185.23 ± 23.51 *	1.56
		brain	136.66 ± 37.23	0.50 ± 0.00	5.11 ± 2.43	2.39 ± 0.15	139.03 ± 15.60	
NBP	I.V.	blood	185.00 ± 15.00	-	2.01 ± 1.53	1.75 ± 0.18	326.65 ± 28.14	0.18
		brain	7.47 ± 2.46	0.58 ± 0.38	0.63 ± 0.34	1.54 ± 0.17	11.33 ± 0.22	
	I.P.	blood	29.17 ± 3.00	0.50 ± 0.00	2.46 ± 0.57	2.41 ± 0.11	85.15 ± 9.59	-
		brain	6.96 ± 1.82	0.58 ± 0.38	1.71 ± 1.14	2.20 ± 0.28	16.52 ± 6.13	
	Solution	blood	22.80 ± 6.14	0.50 ± 0.50	1.37 ± 0.66	1.98 ± 0.22	56.25 ± 10.95	1.57
		brain	9.19 ± 3.77	0.25 ± 0.00	1.64 ± 0.22	2.24 ± 0.17	17.15 ± 3.37	
	NE	blood	29.56 ± 32.59	0.83 ± 0.28	1.88 ± 0.39	2.77 ± 0.59	48.72 ± 0.60	1.87
		brain	6.60 ± 1.67	0.33 ± 0.14	2.92 ± 0.94	2.10 ± 0.37	17.69 ± 4.61	
	NE-ISG	blood	35.66 ± 3.85	1.00 ± 0.00	4.43 ± 2.55	2.22 ± 0.25	50.71 ± 11.03	2.01
		brain	18.33 ± 0.57 * #	0.50 ± 0.00	4.03 ± 1.87	2.92 ± 1.59	19.74 ± 6.36	

Note: *p < 0.05 vs. intravenous group; # *p* < 0.05 vs. gavage group.

## Data Availability

Data are contained within the article and Supplementary Materials.

## Supplementary Materials

The following supporting information can be downloaded at: https://www.mdpi.com/article/10.3390/molecules27217644/s1, Figure S1: Gel strength measuring device. Figure S2: Bioadhesive force-measuring device. Figure S3: Solubility of VOC in different surfactants and cosurfactant. Figure S4: Pseudo ternary phase diagram. Figure S5: 3D surface and interaction plots showing the effects of the independent variables on particle size of VOC-NE. Figure S6: 3D surface and interaction plots showing the effects of the independent variables on zeta potential of VOC-NE. Figure S7: The volite oil of Chaxiong nanoemulsion. Figure S8: Identification results of VOC-NE by staining (left: Sudan red III; right: methylene blue). Figure S9: The particle size distribution and potential distribution of VOC-NE. Figure S10: Effect of P407 concentration on the gelling temperature of in situ gels. Figure S11: The effect of P188 concentration on gelation temperature. Figure S12: The effect of P407 and P188 concentration on gelation temperature of VOC-NE-ISG. Figure S13: Liquid-solid transition state of VOC-NE-ISG (left: liquid state; right: gel state). Figure S14: Rheological evaluation of VOC-NE-ISG. Figure S15: In vitro release curve of VOC-NE-ISG. Figure S16: The MCAO model rats. Table S1: Formulation of Compound Emulsifier. Table S2: Variables and their levels in the Box-Behnken design. Table S3: MS detection conditions for the five components of VOC. Table S4: Effects of surfactants on nanoemulsions. Table S5: Summary of results of regression analysis for the considered responses Y1–Y3. Table S6: Predicted versus observed values of optimized VOC-NE. Table S7: Fitting results of in vitro release model of VOC-NE-ISG (*n* = 3, X ± SD). Table S8: Pharmacodynamic evaluation of VOC-NE-ISG. (*n* = 10, X ± SD).
