# Supplementary material for: Preparation, In Vitro and In Vivo Evaluation of Nanoemulsion In Situ Gel for Transnasal Delivery of Traditional Chinese Medicine Volatile Oil from Ligusticum sinense Oliv.cv. Chaxiong
Source: Molecules. 2022 Nov 7;27(21):7644. doi: 10.3390/molecules27217644 (PMC9655233; doi:10.3390/molecules27217644)
Supplement: Supplementary file 1 [file molecules-27-07644-s001.zip › molecules-2003863-supplementary.pdf]

Supplementary Materials

Tables

Table S1

Formulation of Compound Emulsifier

HLB_{mix}	Tween80（%）	Span80（%）
4.3	0	100
9	44	56
10.5	58	42
12	72	28
13.5	86	14
16	100	0

Table S2

Variables and their levels in the Box-Behnken design.

	Levels		
	-1	0	1
Independent variables			
A=ratio of oil (%)	4	10	16
B=ratio of Smix (%)	20	25	30
C=stirring time (h)	0.5	1.0	1.5
Dependent variables	Constraints		
Y1=mean particle size (MPS, nm)	Minimize		
Y2= polymer dispersity index (PDI)	Minimize		
Y3=zeta potential (ZP, mV)	Minimize		

Table S3

MS detection conditions for the five components of VOC

component	$[M+H]^+$ (m/z)	DP(V)	CE(eV)	Retention time (min)
NBP	191.3→116.9	76	29	4.14
SA	193.1→136.8	76	17	3.96
ZL	191.1→145.0	72	20	5.05
BP	189.3→128.0	76	35	5.13
NOL	195.0→149.0	79	14	4.78
DL（IS）	231.0→185.1	89	18	5.49

Table S4

Effects of surfactants on nanoemulsions

**Surfactant Type**	**traits**	**Size/nm**
Span 80	cloudy, layered	-
Tween 80	cloudy	Yellow-white lotion
RH40	clear, transparent	23.11±0.12

Compound emulsifier 1 （HLB=9）	cloudy	Yellow-white lotion
Compound emulsifier 2 （HLB=10.5）	cloudy	Yellow-white lotion
Compound emulsifier 3 （HLB=12）	cloudy	Yellow-white lotion
Compound emulsifier 4 （HLB=13.5）	cloudy, layered	Yellow-white lotion

Table S5

Summary of results of regression analysis for the considered responses Y1-Y3.

Factors	MPS(Y1)		PDI(Y2)		ZP(Y3)	
	Coefficient	*P*-value	Coefficient	*P*-value	Coefficient	*P*-value
Model	8	0.006*	1.89	0.2073	6.15	0.0129*
A	26.8	0.0013*	4.82	0.0641	1.45	0.2681
B	0.08	0.7857	1.85	0.2154	12.92	0.0088*
C	8.09	0.0249*	1.12	0.3259	1.04	0.3427
AB	6.12	0.0426*	0.26	0.6251	17.71	0.004*
AC	3.2	0.1166	0.006	0.9395	8.77	0.0211*
BC	2.95	0.1297	0.15	0.706	7.55	0.0286*
A^2	0.29	0.6078	2.1	0.1909	2.43	0.1627
B^2	8.4	0.0231*	0.11	0.7535	2.6	0.1509
C^2	14.27	0.0069*	6.89	0.0341*	1.11	0.3268
Lack of Fit	6.29	0.0539	4.59	0.0875	5.27	0.0711
R^2	0.9113		0.7081		0.8877	

Note:*significant value, with a 95% confidence interval. R^2 is the correlation coefficient.

Table S6

Predicted versus observed values of optimized VOC-NE.

**Dependent variables**	**Predicted**	**Observed**	**Prediction error (%)**
Y1=mean particle size (nm)	21.38	21.02±0.17	1.71
Y2=polymer dispersity index	0.14	0.15±0.02	6.67
Y3=zeta potential (mV)	-20.73	-20.40±1.47	1.60

Table S7

Fitting results of in vitro release model of VOC-NE-ISG (n=3, X±SD)

Model	Zero	First	Higuchi	Ritger-peppas
SA	Y=7.37+3.61x<br>R^2=0.9356	lnY=94.89+0.08x<br>R^2=0.9800	$Y = 18.31\sqrt{x} - 7.58$<br>R^2=0.9852	$Y=10.77x^{0.66}$<br>R^2=0.9973
NOL	Y=8.20+3.12x<br>R^2=0.9607	lnY=90.57+0.07x<br>R^2=0.9746	$Y = 15.61\sqrt{x} - 4.23$<br>R^2=0.9709	$Y=10.07x^{0.64}$<br>R^2=0.9856
NBP	Y=8.99+3.17x	lnY=93.34+0.07x	$Y = 15.78\sqrt{x} - 3.51$	$Y=10.66x^{0.63}$

	$R^2=0.9622$	$R^2=0.9627$	$R^2=0.9659$	$R^2=0.9808$
ZL	$Y=4.50+3.11x$<br>$R^2=0.9531$	$\ln Y=96.72+0.05x$<br>$R^2=0.9765$	$Y = 15.47\sqrt{x} - 7.73$<br>$R^2=0.9542$	$Y=7.17x^{0.74}$<br>$R^2=0.9855$
BP	$Y=4.98+280x$<br>$R^2=0.9551$	$\ln Y=91.90+0.05x$<br>$R^2=0.9716$	$Y = 13.87\sqrt{x} - 5.92$<br>$R^2=0.9718$	$Y=6.70x^{0.72}$<br>$R^2=0.9846$

Table S8

Pharmacodynamic evaluation of VOC-NE-ISG. (n=10, $\bar{X}\pm SD$)

group	n	Neurological score after MCAO modeling	Post-dose neurological score	infarct size/%
Sham	10	0.00±0.00	0.00±0.00	0.00±0.00
MCAO	10	2.17±0.72	2.50±0.50	41.36±4.83
MCAO+NBP	10	2.70±0.67	1.20±0.60*	23.02±2.30**
MCAO+NE	10	2.33±0.78	1.60±0.92	31.65±2.60*
MCAO+NE-ISG	10	2.38±0.65	1.30±0.78*	25.14±4.26**

Note: *$P<0.05$, **$P<0.01$ VS model group.

Figures

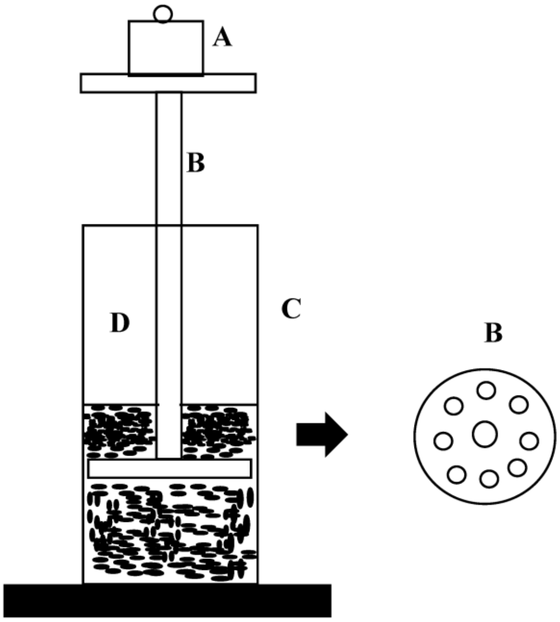

Figure S1. Gel strength measuring device; (A) weigths; (B) device; (C) mess cylinder; (D) poloxamer gel

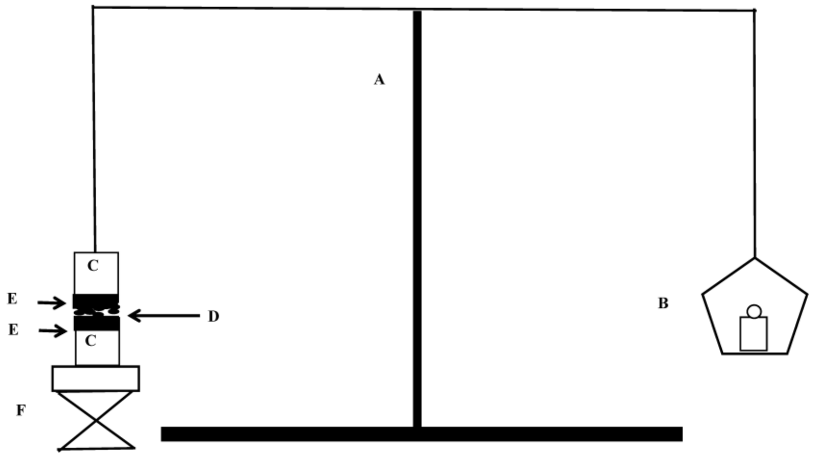

Figure S2. Bioadhesive force-measuring device, (A)modified balance; (B)weights; (C)glass vial; (D)poloxamer gel; (E)nasal mucosa; (F)height-adjustable pan.

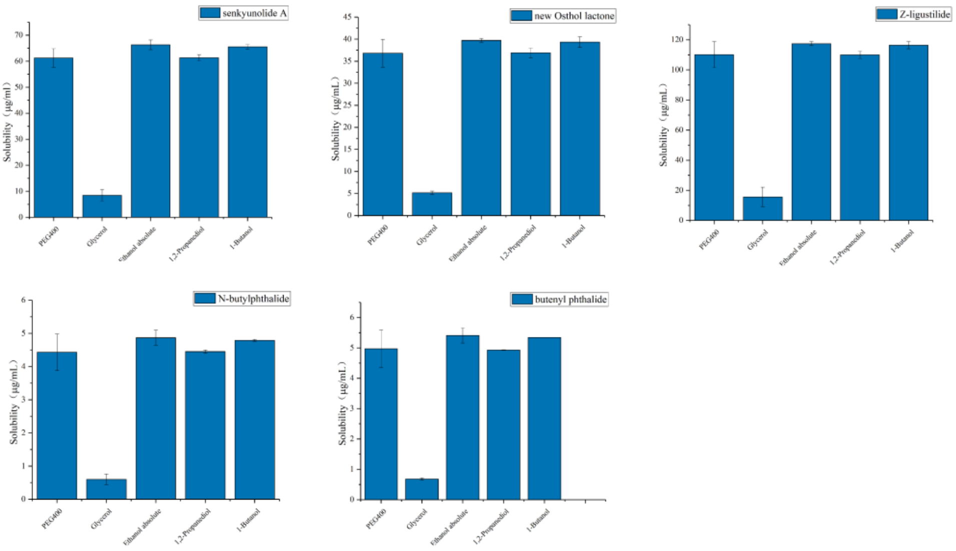

Figure S3. Solubility of VOC in different surfactants and cosurfactants.

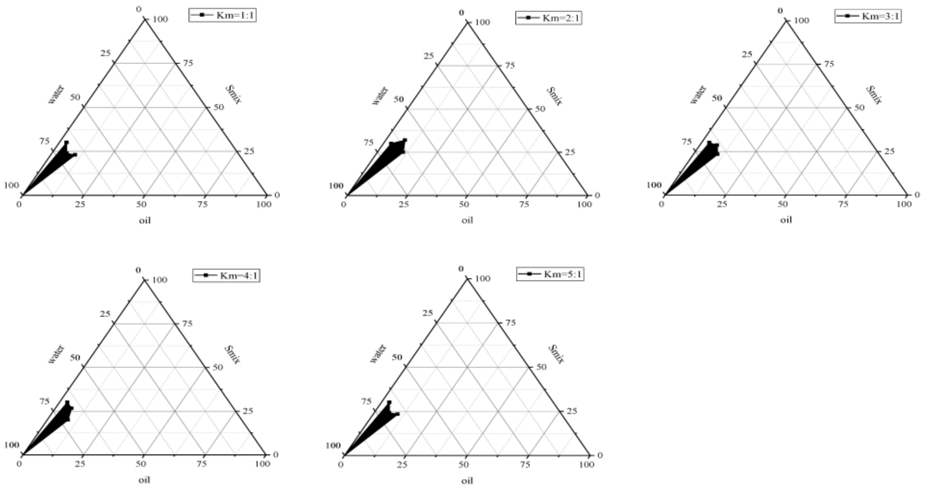

Figure S4. Pseudo ternary phase diagram.

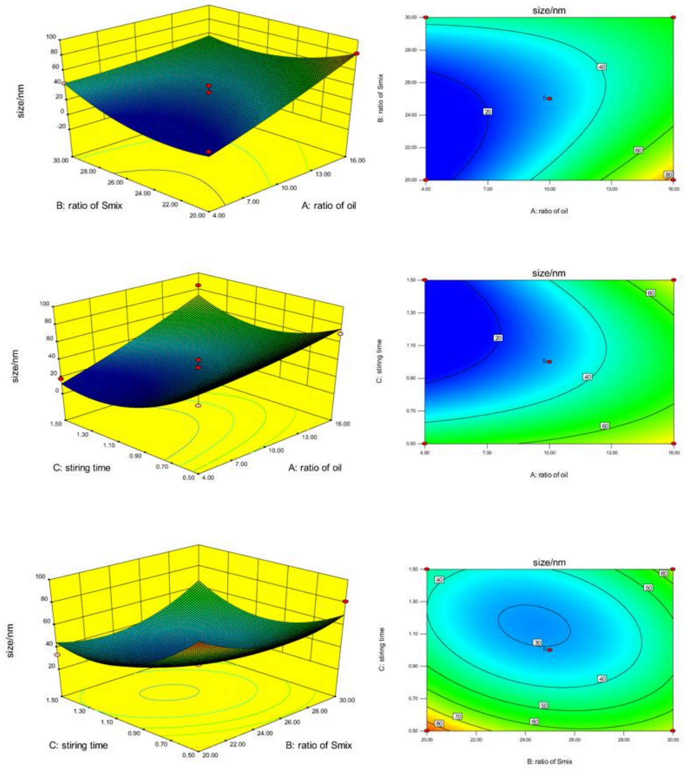

Figure S5. 3D surface and interaction plots showing the effects of the independent variables on particle size of VOC-NE.

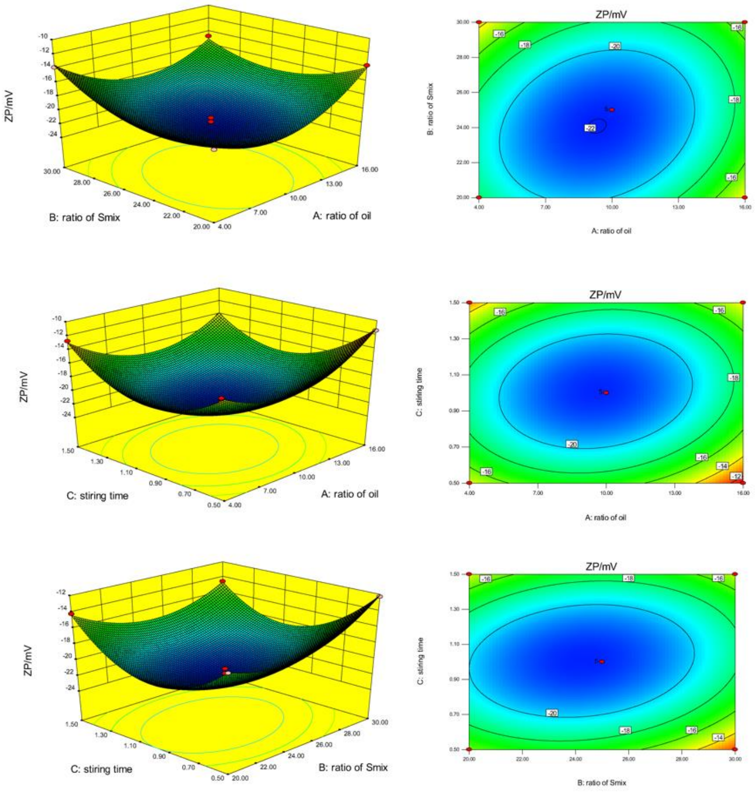

Figure S6. 3D surface and interaction plots showing the effects of the independent variables on zeta potential of VOC-NE.

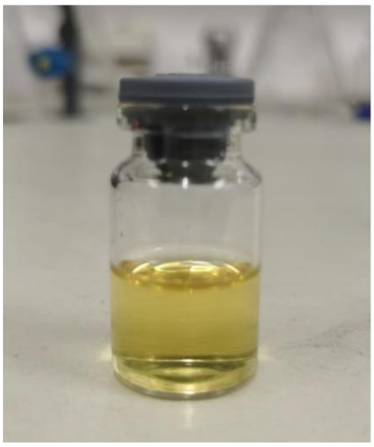

Figure S7. The volite oil of Chaxiong nanoemulsion

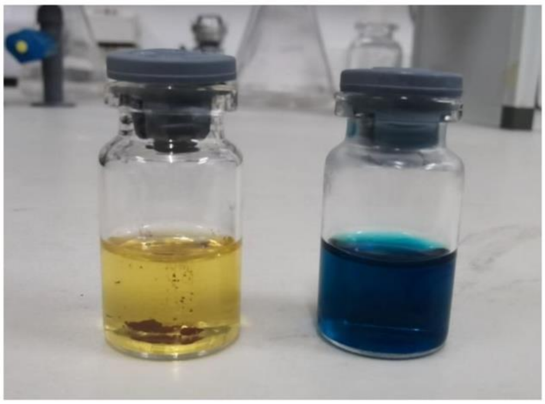

Figure S8. Identification results of VOC-NE by staining (left: Sudan red III; right: methylene blue)

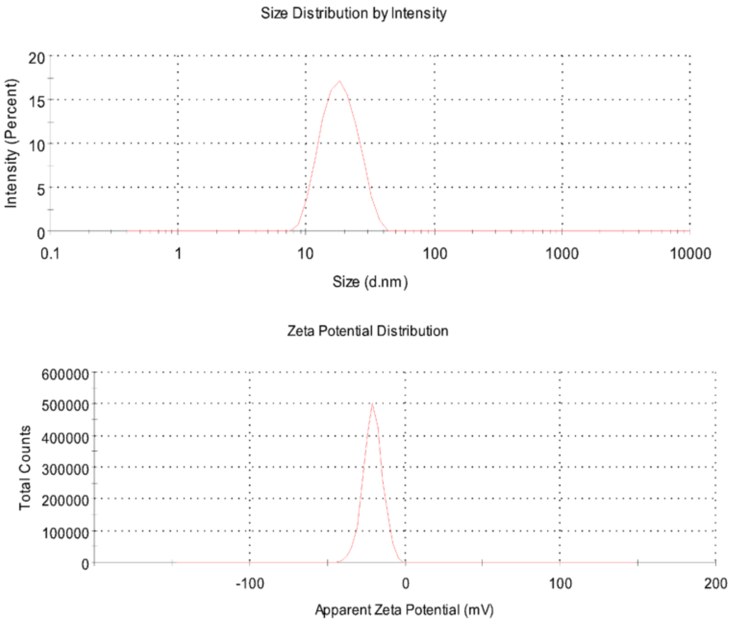

Figure S9. The particle size distribution and potential distribution of VOC-NE.

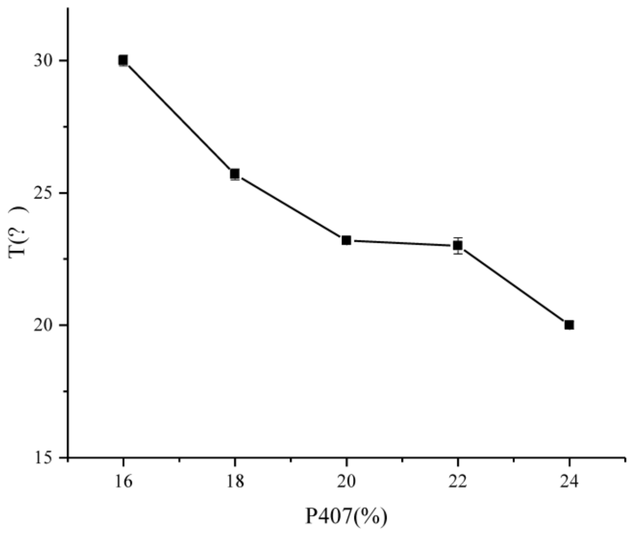

Figure S10. Effect of P407 concentration on the gelling temperature of in situ gels

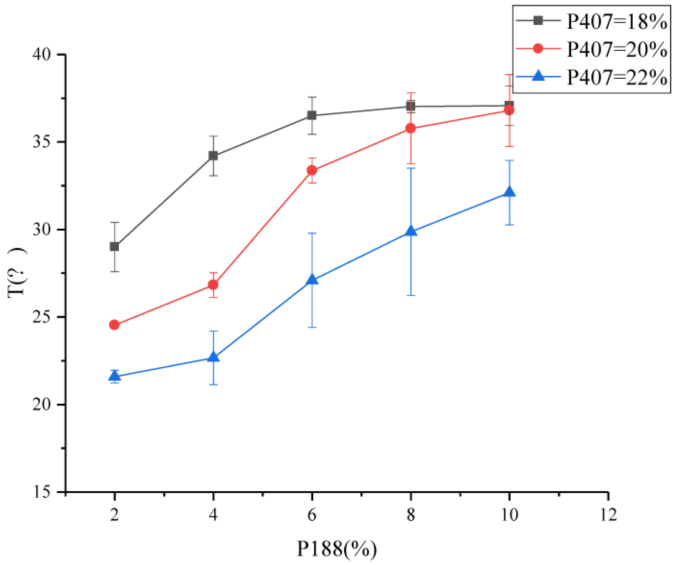

Figure S11. The effect of P188 concentration on gelation temperature

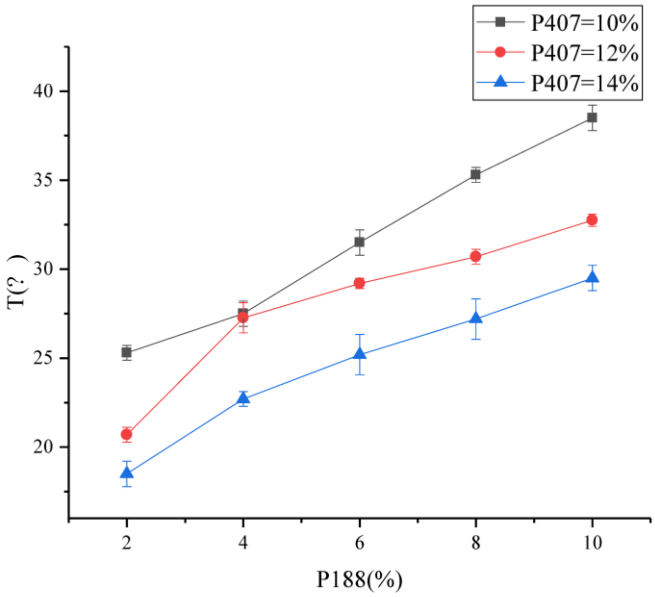

Figure S12. The effect of P407 and P188 concentration on gelation temperature of VOC-NE-ISG

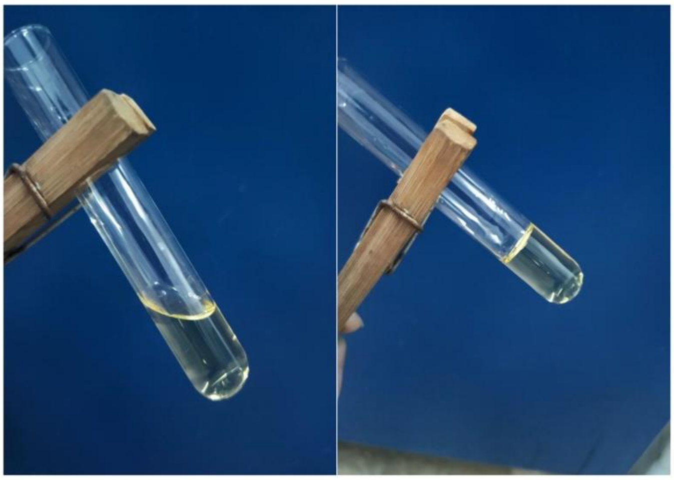

Figure S13. Liquid-solid transition state of VOC-NE-ISG (left: liquid state; right: gel state)

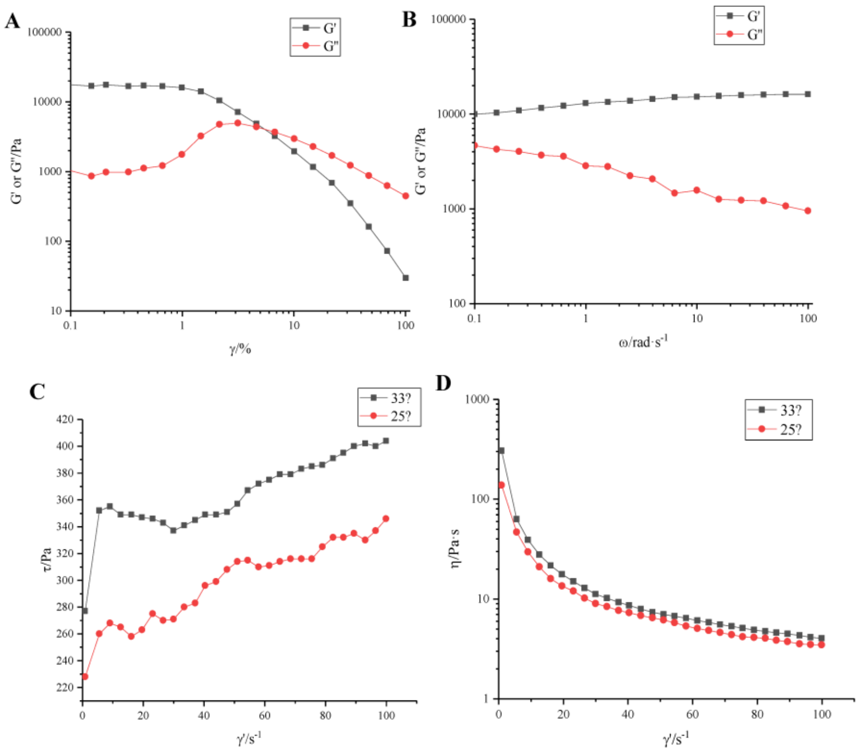

Figure S14. Rheological evaluation of VOC-NE-ISG

A. Linear viscoelastic interval diagram of VOC-NE-ISG B. Frequency scanning curves of VOC-NE-ISG C. τ change curves of CPTA-NE-ISG as a function of γ' at different temperature. D. ηchange curves of CPTA-NE-ISG as a function of γ' at different temperature.

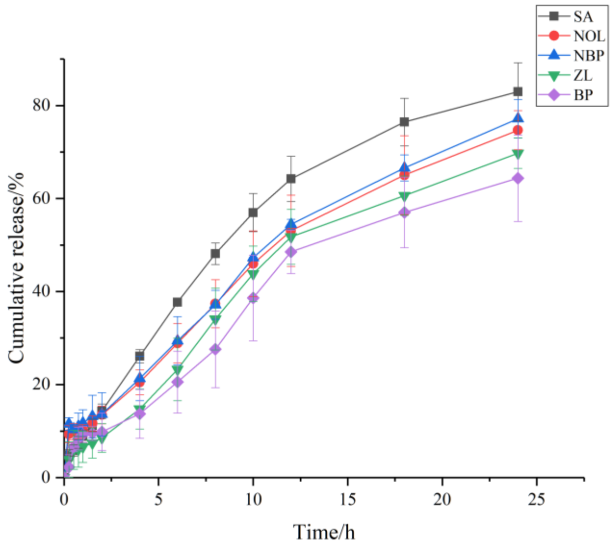

Figure S15. In vitro release curve of VOC-NE-ISG

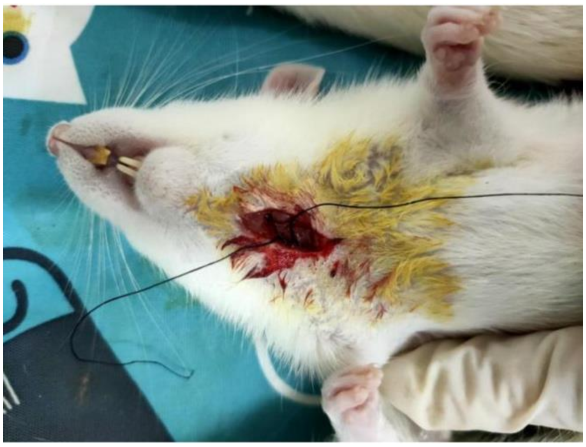

Figure S16. The MCAO model rats
